# Genomic Evidence for Sensorial Adaptations to a Nocturnal Predatory Lifestyle in Owls

**DOI:** 10.1093/gbe/evaa166

**Published:** 2020-08-08

**Authors:** Pamela Espíndola-Hernández, Jakob C Mueller, Martina Carrete, Stefan Boerno, Bart Kempenaers

**Affiliations:** e1 Department of Behavioural Ecology and Evolutionary Genetics, Max Planck Institute for Ornithology, Seewiesen, Germany; e2 Department of Physical, Chemical and Natural Systems, Universidad Pablo de Olavide, Sevilla, Spain; e3 Sequencing Core Facility, Max Planck Institute for Molecular Genetics, Berlin, Germany

**Keywords:** night-active, raptor, genome-wide analysis, comparative genomics, positive selection, Strigiformes

## Abstract

Owls (Strigiformes) evolved specific adaptations to their nocturnal predatory lifestyle, such as asymmetrical ears, a facial disk, and a feather structure allowing silent flight. Owls also share some traits with diurnal raptors and other nocturnal birds, such as cryptic plumage patterns, reversed sexual size dimorphism, and acute vision and hearing. The genetic basis of some of these adaptations to a nocturnal predatory lifestyle has been studied by candidate gene approaches but rarely with genome-wide scans. Here, we used a genome-wide comparative analysis to test for selection in the early history of the owls. We estimated the substitution rates in the coding regions of 20 bird genomes, including 11 owls of which five were newly sequenced. Then, we tested for functional overrepresentation across the genes that showed signals of selection. In the ancestral branch of the owls, we found traces of positive selection in the evolution of genes functionally related to visual perception, especially to phototransduction, and to chromosome packaging. Several genes that have been previously linked to acoustic perception, circadian rhythm, and feather structure also showed signals of an accelerated evolution in the origin of the owls. We discuss the functions of the genes under positive selection and their putative association with the adaptation to the nocturnal predatory lifestyle of the owls.

**Significance**

Beneficial mutations are fixed by positive selection, and the process can be analyzed by comparing genome sequences of different related species. Here, we aim to trace signals of positive selection in the early history of owls. The owls are the only nocturnal raptors among birds with specific adaptations such as acute vision and hearing and silent flight. The genetic basis of these adaptations has been studied in single candidate genes but rarely with genome-wide scans. We found traces of positive selection in the early evolution of owls mostly in genes that are functionally related to visual and acoustic perception.

## Introduction

The owls (Strigiformes) are the only avian lineage of nocturnal raptors. They separated from their sister group, the diurnal Coraciimorph clade, in the Paleocene (Prum et al. 2015), and divided into two families, Strigidae and Tytonidae (Wink et al. 2009; Ponder and Willette 2015). Presumably, the past diversification of owls was associated with a concurrent radiation of small mammals, which led to an expansion of prey availability in the nocturnal niche (Feduccia 1999). Owls evolved an interesting set of raptorial adaptations to the nocturnal niche. Some of those adaptations are shared with other diurnal raptors, whereas others are shared with nocturnal bird species that are not raptors.

Like other raptors, owls have cryptic plumage coloration, reversed sexual size dimorphism as well as acute vision and hearing (del Hoyo et al. 1999; Duncan 2013). With other nonraptor nocturnal birds, such as kiwis and oilbirds, owls share an enhanced visual sensitivity but lost color discrimination to some extent (Martin et al. 2004; Corfield et al. 2015; Emerling 2018). Owls have binocular vision, large tubular eyes, and a duplex retina dominated by rods that characterize a typical nocturnal eye (Walls 1942; Fite 1973). Owls also have unique traits that are clearly adaptive for nocturnal raptors. For instance, many species have asymmetrical ears and a facial disk, which improves their ability to find prey in darkness by hearing (Payne 1971). Additionally, the feathers of owls have a serrated leading edge, a fringe trailing edge, and very fine barbules compared with other birds (Sagar et al. 2017). These features make the feathers softer and allow silent flight (Kopania 2016; Sagar et al. 2017), which presumably also improves hunting success.

Independent of timing of activity, a raptorial lifestyle may involve adaptations for hunting, including visual acuity and forward-looking eyes, claws, and curved beaks. It is likely that these adaptations have been retained among landbirds (Telluraves) from their common raptorial ancestor (Hackett et al. 2008; Jarvis 2014; Prum et al. 2015; McClure et al. 2019). Expected adaptations of diurnal raptors are likely related to the maintenance of the visual system and photoresponse recovery (Wu et al. 2016), blood circulation, nervous system development, olfaction, and beak development (Zhan et al. 2013; Wu et al. 2016; Zhou et al. 2019).

A nocturnal lifestyle generally involves adaptations related to the sensory system, circadian rhythms, and plumage color patterns. For example, previous studies reported associations between diel activity patterns and eye shape and size (Hall and Ross 2007; Lisney et al. 2012), size of olfactory bulbs (Healy and Guilford 1990), neural visual pathways (Gutiérrez-Ibáñez et al. 2013), and iris coloration (Passarotto et al. 2018). In birds, the genetic basis for nocturnal adaptations has mostly been studied in the visual system of two nocturnal species, the kiwi *Apteryx mantelli* and the barn owl *Tyto alba* (Borges et al. 2015, 2019; Le Duc et al. 2015; Emerling 2018). Le Duc et al. (2015) showed that adaptations to nocturnality in kiwis are associated with an increase in the olfactory receptor repertoire and an accumulation of evolutionary changes in genes related to color vision, mitochondrial function, and energy expenditure. The avian visual system is characterized by tetrachromatic vision and dense retinas (Yokoyama 2000; Bowmaker 2008; Davies et al. 2012) and relatively large eyes (Howland et al. 2004; Hall and Ross 2007). The avian retinas have six classes of photoreceptor cells: one rod, four single cones, and one double cone (Hart and Hunt 2007; Bowmaker 2008). The membranes of these photoreceptors contain specific photopigments, that is, light-sensitive molecules formed by an opsin and a chromophore. The opsins can be divided into five subfamilies: visual opsins, melanopsins, pineal opsins, vertebrate nonvisual opsins, and photoisomerases (Terakita 2005; Lamb et al. 2007). The visual opsins trigger the phototransduction cascade after light stimulation in the membrane of photoreceptor cells. Cones and rods use different sets of opsins and phototransduction molecules and are specialized in photopic (bright light conditions) and scotopic (dim light conditions) vision, respectively (Lamb et al. 2016). Thus, the cones provide acute and color vision, and the rods are highly sensitive to light.

Diel activity patterns are highly constrained by phylogenetic history (Anderson and Wiens 2017). The majority of the extant avian species are diurnal, but the diel activity pattern of the common ancestor of all birds is unknown. Two hypotheses have been proposed. The first hypothesis is that the avian common ancestor had a diurnal lifestyle, which is supported by a vast amount of morphological and genetic evidence (Schmitz and Motani 2011; Anderson and Wiens 2017). For instance, the ancestral bird probably had similar color discrimination as the diurnal modern birds, because there is no evidence for any global loss or gain of genes related to color vision among birds (Zhang et al. 2014; Borges et al. 2015). The second hypothesis proposes that the common ancestor was nocturnal, with a transition to cathemeral (active during day and night), similar to mammals (Wu and Wang 2019). Assuming a diurnal common ancestor, nocturnality evolved many times independently in parrots, kiwis, oilbirds, nightjars, and owls (Ericson et al. 2006; Hackett et al. 2008; Braun and Huddleston 2009; Le Duc et al. 2015). This should be paralleled by the accumulation of genetic changes related to nocturnality on each of the ancestral branches of the nocturnal clades.

The genetic basis of adaptations to a nocturnal and raptorial lifestyle has been studied by candidate- gene approaches but rarely with genome-wide scans, and the results have been mostly related to the visual system. Thus, how evolution shaped the specific combination of traits observed in the owls remains poorly understood. Here, we aim to answer the following questions. 1) What is the general role of positive selection in the early adaptive history of Strigiformes? 2) Which genes and associated functions evolved under positive selection in the owls? 3) Are the positively selected genes associated with adaptation to the night-active or the predatory lifestyle of the owls? We used substitution rates to test for selection in the early history of Strigiformes in a genome-wide comparative analysis, using 20 species of birds including 11 owls of which five were newly sequenced for this study. Complementing the search for single, genome-wide significant genes, we used overrepresentation analyses to functionally interpret groups of genes that showed any signal (including weak signals) of positive selection.

## Materials and Methods

### Study Species and Reference Genome

This study includes genomes from 20 bird species that were selected to produce a balanced tree around the ancestral branch of the owls: 11 Strigiformes, two Accipitriformes, four Coraciimorphae, one Falconiform, one Passeriform, and one Galliform. In contrast to the mostly nocturnal Strigiformes, all other species included in this study are diurnal. We included Coraciimorphae as the sister group to the owls, Accipitriformes and Falconiformes as diurnal raptors, and the Passeriformes and Galliformes because of their high genome sequence quality (Zhang et al. 2014).

All genomes included in this study were aligned using the assembly of *Athene cunicularia* (burrowing owl, assembly athCun1) as reference genome (Mueller et al. 2018). The burrowing owl is a peculiar species among the owls, being diurnal and gregarious, which implies that its genome may contain some unique features and may lack some of the genes that are present in the rest of the owls. However, drastic gene loss is unlikely considering the short evolutionary history of burrowing owls. Moreover, we used a selection test (“*ω* test”) that is based on the codon sequences that are common among all the compared species, including non-owls. Therefore, the important criteria to avoid loss of information are the assembly completeness and the continuity of the annotated gene sequences to construct the multispecies codon alignment for the selection test (see below). We therefore used athCun1 as the reference, because it is the highest-quality owl genome assembly that was available in terms of completeness and N50 criterion: athCun1 has longer scaffolds, the assembly is more continuous and more complete. The assembly contained 94.8% of complete Benchmarking Universal Single-Copy Orthologs (BUSCO v. 4.0.6) based on the avian database of 8,338 genes (BUSCO summary in supplementary file 1, Supplementary Material online) (Simão et al. 2015). The reference genome was annotated by the NCBI Eukaryotic Genome Annotation Pipeline (NCBI *Athene cunicularia* Annotation Release 100; NCBI Assembly Accession GCA_003259725.1).

### Protocol A: Sequencing and Read Mapping to Reference

The following eight owl genomes were sequenced and mapped to athCun1: *Bubo scandiacus* (snowy owl), *Strix uralensis* (ural owl), *Strix nebulosa* (great gray owl), *Athene noctua* (little owl), *Surnia ulula* (northern hawk-owl), *Bubo bubo* (Eurasian eagle-owl), *Asio otus* (long-eared owl), and *Asio flammeus* (short-eared owl). The DNA was obtained from blood samples stored in ethanol. For the majority of the samples, we extracted the DNA using the QuickPure kit (Macherey-Nagel) applying a predigestion with Proteinase K in Digsol buffer. After initial quality control, we used the Kapa HyperPrep DNA kit (Roche) to prepare 200 to 300 bp insert paired-end libraries. Then, the majority of the samples were sequenced with an Illumina HiSeq4000 in paired-end, 150 bp mode (Sequencing Core Facility of the Max Planck Institute for Molecular Genetics, Berlin, Germany), yielding between 74 and 141 million fragments (read pairs) mapped per individual sample (15.2–26.8× genome coverage). The samples of *Athene noctua*, *Asio otus*, and *Asio flammeus* were extracted using the phenol–chloroform method; the libraries were prepared using Illumina’s TruSeq DNA protocol and sequenced on an Illumina HiSeq2500. After sequencing, we used the aligner software BWA-MEM v.0.7.17-r1188 (Li 2013) to map the reads of each species against the reference genome. Parameters are detailed in section 1.1 of the supplementary file 1, Supplementary Material online.

### Protocol B: Genome-Scale Sequence Mapping to Reference

For the following species, we downloaded the genome assemblies from NCBI (accession numbers and details in supplementary table S1 in file 1, Supplementary Material online): *Strix occidentalis* (spotted owl), *Tyto alba* (barn owl), *Falco peregrinus* (peregrine falcon), *Taeniopygia guttata* (zebra finch), *Picoides pubescens* (downy woodpecker), *Apaloderma vittatum* (bar-tailed trogon), *Leptosomus discolor* (cuckoo roller), *Colius striatus* (speckled mousebird), *Cathartes aura* (Turkey vulture), *Haliaeetus leucocephalus* (bald eagle), and *Gallus gallus* (red junglefowl). We downloaded the assemblies as FASTA files and aligned them to the reference genome using LAST v. 921 (Kiełbasa et al. 2011). Parameters are detailed in section 1.1 of the supplementary file 1, Supplementary Material online. The overlapping regions were resolved with SingleCov2 (Multiz-tba.012109, Blanchette et al. 2004) with default parameters, and the final alignment was created using maf-convert (LAST v. 921) and samtools (Li et al. 2009).

The pairwise sequence alignments produced by both protocols, a and b, were similar among owls in terms of gaps and percentage of the reference genome covered (supplementary table S1 in file 1, Supplementary Material online).

### Multispecies Codon Alignment

After the alignment of each species to the reference, the general workflow consisted of six steps to produce a multispecies codon alignment for each annotated gene in the reference genome (supplementary fig. S1 in file 1, Supplementary Material online). 1) Piling up the reads in the coding regions using samtools. 2) Variant calling with bcftools (Danecek and McCarthy 2017). 3) Producing the consensus sequence using default parameters with bcftools, choosing the allele with more reads or better mapping quality in case of heterozygous sites. 4) Masking all the sites with zero read coverage. Note that the species with lower read coverage or those more distantly related to the reference had a higher percentage of masked sites (see supplementary table S1 in file 1, Supplementary Material online). 5) Extracting the sequence of each gene from the consensus sequence of each species and concatenate all in a single, multispecies FASTA file with bedtools (Quinlan and Hall 2010; Dale et al. 2011). 6) Running a multispecies codon alignment for each gene using MACSE (Ranwez et al. 2011). We used MACSE because it aligns protein-coding gene sequences correcting for potentially erroneous frameshifts (e.g., indels smaller than triplets) without disrupting the underlying codon structure.

Finally, we inferred the percentage of low-quality regions of each multispecies gene alignment using BMGE v. 1.12 (Criscuolo and Gribaldo 2010). After removing codon sites with missing data (gaps) in one of the species (similar to the procedure of the *ω* tests; see below) we identified sites with a smoothed entropy-score higher than 0.5. These highly variable regions were considered as low-quality regions, potentially caused by misalignments or sequencing errors (small indels). Genes (multispecies alignments [MSAs]) with any low-quality region were excluded for further analyses (*N* = 10 genes). We used AMAS (Borowiec 2016) to quantify the percentage of variable sites.

### Phylogenetic Tree

**Fig. 1.**
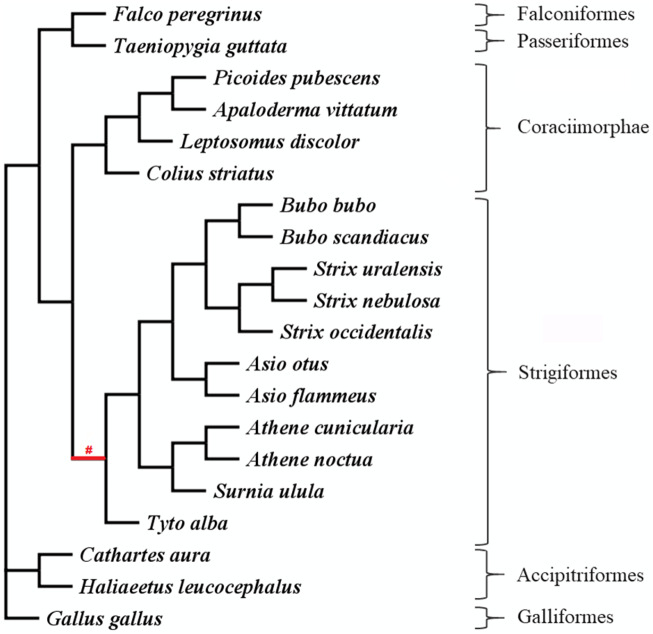
Unrooted species tree without branch lengths extracted from Prum et al. (2015) and Wink et al. (2009). The *ω* tests were based on this tree, whereby the red symbol “#” indicates the foreground branch in contrast to the rest of the branches (background).

The phylogenetic tree of the selected species was based on information from the phylogeny of all birds (Prum et al. 2015) and the phylogeny of owls (Wink et al. 2009). The subset of species was extracted, keeping the topology and ignoring the branch lengths, using the software Mesquite version 3.40 (Maddison W and Maddison D 2018). Figure 1 shows the unrooted tree used for the selection tests in CodeML.

### Selection Tests

To assess past selection on each gene at the ancestral branch of the owls, we estimated the nonsynonymous to synonymous substitution rate ratio (*ω* = d*N*/d*S*; for a review, see Nielsen [2005]). This ratio measures the direction and magnitude of selection on protein-coding genes. In the rest of the text, we simply refer to it as the “*ω* test” (for the test) and the “*ω* value” (for the estimated value).

The *ω* value of each gene can be calculated for specific branches of a phylogenetic tree and reflects the evolutionary history of that branch, with *ω* < 1 indicating purifying selection, *ω* = 1 neutral evolution, and *ω* > 1 positive selection. We tested *ω* for the ancestral branch of Strigiformes using a maximum-likelihood method implemented in the CodeML program in PAML 4.9h (Yang 2007), using the branch model (Yang 1998) and the branch-site model (Yang and Nielsen 2002; Zhang et al. 2005; Yang and dos Reis 2011). For both models, we excluded all the sites with missing data in the MSA, and we defined the ancestral branch of Strigiformes as the foreground (label “#” in fig. 1).

When a gene had a *ω* value >1 in the ancestral branch of the owls, we considered this as evidence of strong positive selection, that is, the nucleotide changes in this gene were adaptive for the ancestral owl. When a gene has a *ω* value that lies between the background value and 1 (*ω*_background_ < *ω*_foreground_ < 1), two interpretations are possible. First, it can indicate relaxed purifying selection, suggesting a loss of function of that gene. Second, it can indicate weak positive selection acting only in a few sites or for a short period. We cannot distinguish between these two options. The majority of the protein-coding sequences are conserved during most of their evolutionary history (*ω*  < 1), but positive selection acting only in few sites in the foreground branch would increase the average *ω* value of the foreground above the background (Toll-Riera et al. 2011; Nery et al. 2013). We identified genes with *ω* values <1, but with a significantly higher value on the ancestral branch of the owls than in the background in a separate category of “weak positive selection or relaxed purifying selection.” We also used the term “accelerated substitution rate” to concisely describe the *ω* values of genes in this category in combination with the category of “strong positive selection.”

The branch model tests a null hypothesis (H_0_), assuming all branches of the phylogenetic tree have the same *ω* ratio, against an alternative hypothesis (H_1_), where the labeled branch of interest (“foreground”) has a different *ω* ratio (*ω*_1_) than all other branches of the phylogenetic tree (*ω*_0_, “background”).

The branch-site model tests for positive selection among codon sites in the ancestral branch of the owls. In this model, *ω* is allowed to vary between foreground and background branches as well as among sites on each gene, under both the null (H_0_) and the alternative hypothesis (H_1_). This represents a more realistic and complex scenario where different codon sites of the same gene can evolve under different selection, and selection can also differ among the branches in the phylogeny. The model estimates the proportion of sites that have certain combinations of *ω* values for the foreground and background. The estimated foreground and background *ω* values for each site are then divided into three categories: *ω* < 1, *ω* = 1, and *ω* > 1 (referred to as *ω*_0_, *ω*_1_, and *ω*_2_). Under the H_0_, no *ω* is allowed to be larger than 1, both in the foreground and the background, meaning that positive selection is not allowed at any site. Under the (H_1_) , some *ω* values (at some sites) can be larger than one in the foreground branch, representing the category of positively selected sites (for a summarized explanation of this model see https://selectome.unil.ch/cgi-bin/methods.cgi, last accessed August 19, 2020). Thus, this model tests a null hypothesis (H_0_), where the foreground cannot have positive selection at any site, against an alternative hypothesis (H_1_), where the foreground lineage is allowed to have a proportion of sites evolving under positive selection (Yang and Nielsen 2002; Yang and dos Reis 2011).

For each model, we tested whether the alternative hypothesis is more likely than the null hypothesis using the likelihood ratio test (LRT) statistic, that is, twice the difference in log-likelihoods between the two hypotheses (LRT = 2 × [ln L_H1_ – ln L_H0_]), which is compared with a *χ*^2^ distribution with one degree of freedom. Hence, the LRT was considered significant when >3.8415. We excluded genes with significant LRT values but estimated foreground *ω* values >500 (24% of genes for the branch model and 40% of genes for the branch-site model) from further analyses, because such high *ω* values in CodeML indicate a synonymous substitution rate estimate close to 0, which means that *ω* cannot be reliably calculated (Yang and dos Reis 2011). The results of all genes with nominal significant *ω* tests and of all a priori defined candidate genes (including nonsignificant results) are in supplementary file 2, Supplementary Material online.

We identified 27,746 annotated isoforms for the protein-coding genes in the athCun1 reference genome. We applied filters to these annotated isoforms before and after the tests, to select gene sequences that fulfilled the requirements for the *ω* tests and further functional analyses. First, we selected the longest isoform for each protein-coding gene (13,841 unique genes) with at least 20 codons in the MSAs and with at least one variant site, and without regions of potential misalignment problems as measured by high-entropy blocks. This yielded 12,160 genes for the *ω* tests. Second, we filtered out the genes with estimated *ω* values >500 on the ancestral branch of the owls (*N* = 629 genes for the branch model and 231 for the branch-site model). After the filters, we applied a false-discovery-rate (FDR) correction for multiple testing for each model. The raw and corrected *P* values are included in supplementary file 2, Supplementary Material online. For further analyses, we considered three categories of genes with a significant *ω* test: i) those showing strong positive selection signals according to the branch model, ii) those with weak positive or relaxed purifying selection according to the branch model, and iii) those with positive selection according to the branch-site model. We refer to these as list i, ii, and iii.

As an alternative to the branch-site model, we applied the aBSREL model (Smith, 2015) of the HyPhy package to all nominal significant genes from the branch-site model (list iii) to search for selection signals specific for owls. We ran this model with two settings: with and without the a priori specified foreground. The first setting is similar to the one used in CodeML and we used it to compare the CodeML results. The second setting explores all the branches of the tree and then selects the genes that have a significant signal in the ancestral branch of the owls, but not in any other branch. The significance threshold is corrected for the number of branches tested.

Multinucleotide mutations within codons are known to cause false inferences of the branch-site model (Venkat et al. 2018). Thus, we quantified the proportion of codons with multiple differences (CMDs) between owls and chicken and used this measure as a proxy for codons with multiple substitutions in the ancestral owl branch. First, we read each MSA as a matrix using R 3.5.0 (R Core Team 2018) and the R package “ape” (Paradis et al. 2004;Paradis and Schliep 2019). Using the MSA as a matrix allows us to compare the nucleotide sites between each owl species and the chicken sequence. Starting from the first nucleotide, every three consecutive nucleotides are considered a codon site. A codon site of the alignment that contained more than one difference for any of the owl species with the chicken sequence was counted as one CMD. We tested whether there is an effect of the proportion of CMDs on the outcome of the *ω* tests (*t*-test) and estimated *ω* values (correlation) for the branch-site model (supplementary fig. S2*a* and *b* in file 1, Supplementary Material online). We applied a two-sample permutation *t*-test using the R package “Deducer” (Fellows 2012) and a Kendall’s rank correlation test using the R package “base.” We found an effect on the significance level (Welsh *t*-statistic, *t* = −9.084, *P* value < 0.001), but no effect on the estimated *ω* values (*τ*_B_ = 0.0096, *P* value = 0.22) in the branch-site model (supplementary fig. S2, Supplementary Material online).

### Overrepresentation Analysis

We performed overrepresentation analyses to test whether the gene sets of the three *ω* test categories (list i–iii) and the genome-wide category were enriched for a particular biological function or a metabolic pathway. We used two software packages for this analysis: ClueGO v2.5.4 plug-in (Bindea et al. 2009) in Cytoscape (Shannon et al. 2003) and the R package GOfuncR (Grote 2019). Both packages use a standard candidate-versus-background hypergeometric enrichment test with a custom functional annotation database as the background gene list. We made this custom annotation database for all genes occurring in the reference athCun1, combining human (org.Hs.eg.db) and chicken (org.Gg.eg.db) annotations of gene ontologies (GOs) and KEGG pathways (Huber et al. 2015; Pagès et al. 2019). 

ClueGO reduces the redundancy among the GO terms by grouping the significantly enriched GO terms based on the shared genes (Bindea et al. 2009). Each functional group in the graph has a leading GO term, which is the most significant term. ClueGO uses a Bonferroni correction for multiple testing by using the number of genes in the gene sets as a proxy for the number of tests. GofuncR uses a more conservative method for multiple testing correction, using family-wise error rates (FWERs) based on 10,000 random permutations of the gene-associated variables (candidate-versus-background genes). In general, the results were consistent between the two methods.

### Candidate Genes Related to the Nocturnal Predatory Lifestyle of Owls

In addition to the data-driven, genome-wide approach, we specifically tested for positive selection on an a priori defined list of candidate genes that are likely related to the nocturnal predatory lifestyle of owls based on previous studies (information-driven or candidate-gene approach). We used 1) genes proposed as candidates by previous studies (Le Duc and Schöneberg 2016; Wu et al. 2016) and 2) genes found by key-word searching on GO terms in AmiGO2 (Carbon et al. 2009). The included keywords were: vision, eye, ear, hearing, vestibular (because the vestibular system is part of the inner ear and brings balance and spatial orientation), circadian, sleep (pooled together with the circadian genes), and keratin (as feathers are made of β-keratins). We identified 253 candidate genes in the reference assembly athCun1, listed in supplementary table S2 of the file 1, Supplementary Material online, in the following four categories: vision (*N* = 104), hearing (*N* = 69), circadian rhythm (*N* = 67), and feather keratin (*N* = 13).

The vision category includes the opsin genes. We searched in athCun1 for all genes in the opsin gene family that are annotated in *Gallus gallus* (galGal5), using BLAST reciprocal best hits with the web-based tool Galaxy (https://usegalaxy.eu/, last accessed July 17, 2020) (Afgan et al. 2018). Nine opsin genes were found in athCun1: *RHO*, *OPN1MSW*, *OPN3* (opsin-3, or encephalopsin), *OPN4* (melanopsin), *OPN4*-1 (melanopsin-like), *OPN5* (opsin-5, or neuropsin), *OPNVA* (vertebrate ancient opsin), *RGR* (retinal G-protein-coupled receptor), and *RRH* (retinal pigment epithelium-derived rhodopsin homolog).

We based our interpretation and discussion of the functions of the relevant candidate genes and the genes that are part of the networks from the overrepresentation analysis on information found in the following databases: NCBI (https://www.ncbi.nlm.nih.gov/search/, last accessed July 17, 2020), GeneCards (Safran et al. 2010) (https://www.genecards.org/, last accessed Juyl 17, 2020), AmiGO2 (Carbon et al. 2009) (http://amigo.geneontology.org/amigo, last accessed July 17, 2020), and reactome (Fabregat et al. 2018).

## Results

### Data-Driven Approach: Genes with Genome-Wide Significant Selection Signals in the Owl Ancestor

After correction for multiple testing across all tested genes, 21 genes of the branch model (list i and ii) and two genes of the branch-site model (list iii) were significant with a 5% FDR (supplementary table S3 in file 2, Supplementary Material online). Considering this set of genes (22 in total from lists i–iii), one GO term, “detection of stimulus involved in sensory perception,” was significantly enriched (FWER = 0.001).

### Data-Driven Approach: Genome-Wide Functional Overrepresentation of Genes with Nominal Significant Selection Signals in the Owl Ancestor

**Fig. 2.**
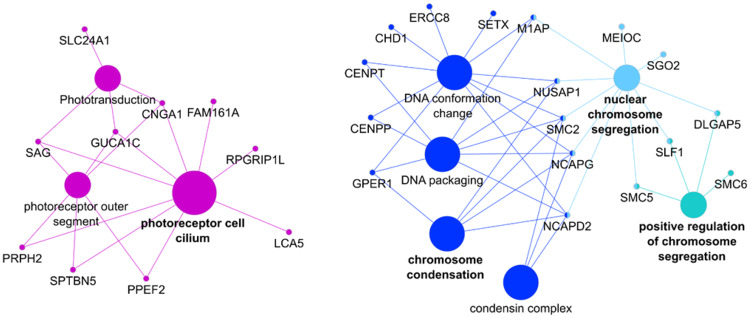
Functional overrepresentation of GO terms and KEGG pathways among the genes with signals of strong positive selection (list i). The GO terms were clustered in four groups by the ClueGO software (shown in different colors). Each group can contain several GO terms with shared genes. There are two major groups: ten genes are related to the visual system (purple group) with “photoreceptor cell cilium” as leading GO term, and 17 genes mostly related to “chromosome condensation” (blue, light blue, and turquoise).

**Fig. 3.**
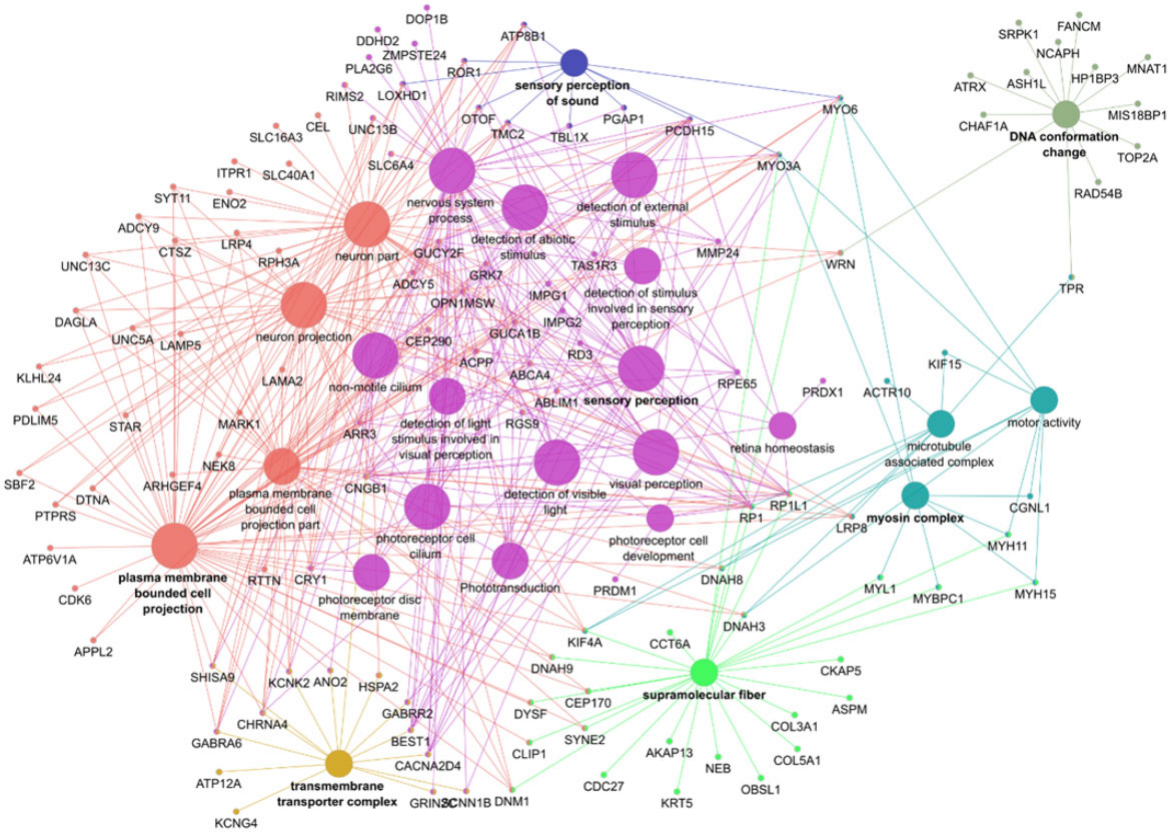
Functional overrepresentation of GO terms and KEGG pathways among the genes with a signal of weak positive or relaxed purifying selection (list ii). The GO terms were clustered in seven groups by the ClueGO software (shown in different colors). Each group can contain several GO terms with shared genes. The groups “plasma membrane bounded cell projection” (salmon) and “sensory perception” (purple) form the main part of the network with 87 genes. This main part also overlaps in several genes with the groups “sensory perception of sound” (blue), “transmembrane transporter complex” (gold), “myosin complex” (turquoise), and “supramolecular fiber” (lime). The functional group “DNA conformation change” with 13 genes forms another, more isolated cluster.

**Fig. 4.**
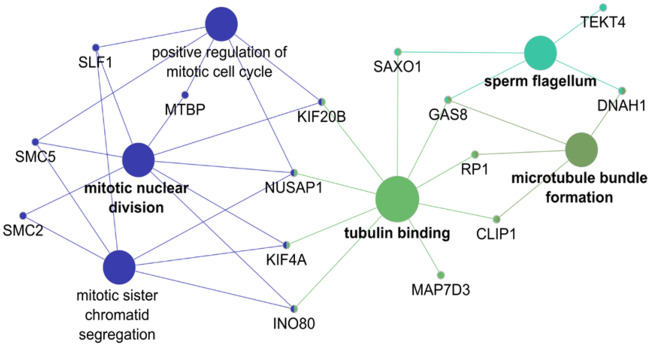
Functional overrepresentation of GO terms and KEGG pathways among the genes that show signals of positive selection on specific sites of the ancestral branch of the owls (list iii, branch-site model). The GO terms were clustered in four groups by the ClueGO analysis (shown as different colors). Each group can contain several GO terms with shared genes. The two major groups are related to “mitotic nuclear division” (blue) and to functions linked to microtubules and tubulin, including sperm flagellum (all other colors). Some genes, such as *RP1* (see also fig. 3), also participate in the development and maintenance of photoreceptors.

The *ω* test based on the branch model was nominal significant for 486 out of 11,613 tested genes (supplementary table S4 in file 2, Supplementary Material online). We differentiated between genes with a signal of strong positive selection on the foreground (list i, with *ω*_0_ ≤ 1 < *ω*_1_; *N* = 199 genes), and genes with a signal of weak positive or relaxed purifying selection on the foreground (list ii, with *ω*_0_ < *ω*_1_ < 1; *N* = 287 genes). The branch-site model identified 123 genes with a signal of positive selection on specific codon sites in the foreground (list iii, with *ω*_2_ > 1; supplementary table S5 in file 2, Supplementary Material online). Supplementary tables S4 and S5 in file 2, Supplementary Material online, show the raw and FDR corrected *P* values of all nominal significant results included in the overrepresentation analysis. We identified 42 genes that were in common between both models (28 shared genes in lists i and iii, and 14 in lists ii and iii). The tests based on the aBSREL model are significant for 59% of the significant branch-site tests (73 tests out of the 123 tested genes in list iii when the model was run with the a priori specified foreground; supplementary table S6 in file 2, Supplementary Material online). After running the model without the a priori specified foreground, we found nine genes with a positive selection signal specific for the ancestral branch of the owls (supplementary table S6 in file 2, Supplementary Material online). The genes with a signal of strong positive selection on the foreground (list i) showed enrichment in four functional groups (fig. 2). These groups form two major networks functionally associated with photoreceptor cells and chromosome condensation. The GO terms that were overrepresented among the genes that evolved under weak positive or relaxed purifying selection in the foreground (list ii) clustered into seven functionally enriched groups (fig. 3). Most of these groups formed a highly connected large network associated with functions of sensory perception (visual and auditory) and plasma membrane bounded cell projection. Another smaller isolated group related to the function of DNA conformation change. The GO terms that were overrepresented among the genes that evolved under positive selection in specific sites of the foreground (list iii) clustered in four functional groups (fig. 4). Most of the functions of these groups are associated with microtubules, including “mitotic nuclear division” and “sperm flagellum.” The detailed results of all overrepresentation analyses with statistical support values are shown in supplementary file 1, Supplementary Material online. Functional groups and single GO/KEGG terms from the overrepresentation analysis by ClueGO are listed in supplementary tables S7 and S8, Supplementary Material online, respectively; supplementary table S9, Supplementary Material online, shows the results of the overrepresentation analysis by GOfuncR. Irrespective of the different multiple testing correction methods, ClueGO and GofuncR produced consistent results.

### Information-Driven Approach: Selection Signals in A Priori Defined Candidate Genes Related to the Nocturnal Predatory Lifestyle of Owls

From the 253 identified candidate genes in the annotation of the reference athCun1, 40 genes had significant *ω* tests, of which 37 were based on the branch model and three on the branch-site model (significant results are in table 1 and results for all candidate genes are in supplementary table S10 of the file 2, Supplementary Material online). Only one candidate gene (*RP1*) showed evidence for selection in both models (lists ii and iii). The total number of significant results is more than expected by chance (253 × 0.05 ∼ 13 expected significant tests).

**Table 1:** Candidate Genes that Evolved under Positive Selection or Relaxed Purifying Selection in the Ancestral Branch of the Owls

Gene Symbol	List	Candidate Gene Category	No. Codons Tested	% of Reference Gene Tested	Branch Model			Branch-Site Model			
					Alternative Hypothesis			Alternative Hypothesis			
					*ω*_0_	*ω*_1_	LRT Statistic	*ω*_0_	*ω*_1_	*ω*_2_	LRT Statistic
ABCA4	ii	Vision	2,236	95.9	0.25	0.66	6.92	0.09	1	1.00	<0.01
ARR3	ii	Vision	305	77.6	0.06	0.85	4.19	0.03	1	1.00	<0.01
ATP8B1	ii	Vision	825	65.5	0.09	0.49	9.13	0.04	1	2.50	0.20
BEST1	ii	Vision	743	97.3	0.09	0.33	4.56	0.03	1	1.00	0.00
CACNA2D4	ii	Vision	963	87.3	0.06	0.71	29.83	0.03	1	3.70	0.96
CNGA1	i	Vision	605	93.7	0.15	1.63	8.73	0.03	1	5.03	0.45
CNGB1	ii	Vision	596	48.1	0.19	0.50	5.62	0.04	1	8.24	1.03
CNGB3	iii	Vision	738	94.6	0.31	0.28	0.02	0.06	1	245.42	9.44
GABRR2	ii	Vision	479	98	0.14	0.73	9.60	0.06	1	3.49	0.56
GRK7	ii	Vision	550	100	0.28	0.72	5.32	0.04	1	2.33	0.45
GUCA1B	ii	Vision	198	100	0.03	0.25	4.66	0.02	1	5.99	0.18
GUCA1C	i	Vision	190	100	0.16	2.87	9.19	0.04	1	3.99	0.34
GUCY2F	ii	Vision	1,115	97.9	0.25	0.51	4.00	0.06	1	1.99	0.12
OPN1MSW	ii	Vision	254	71.5	0.05	0.46	13.65	0.04	1	1.07	<0.01
PCDH15	ii	Vision	2,105	95.7	0.12	0.60	19.63	0.03	1	19.14	1.08
PRPH2	i	Vision	354	100	0.10	1.58	11.20	0.03	1	3.65	0.61
RGS9	ii	Vision	453	93.4	0.12	0.96	8.58	0.03	1	2.99	0.30
RP1	ii and iii	Vision	1,950	92.1	0.42	0.97	5.51	0.16	1	39.69	7.44
RPE65	ii	Vision	514	93.3	0.02	0.11	9.46	0.01	1	4.21	0.87
RRH	i	Vision	334	100	0.11	52.01	6.47	0.05	1	256.52	0.38
SAG	i	Vision	388	95.6	0.26	1.27	8.14	0.06	1	1.00	<0.01
SLC24A1	i	Vision	615	92.1	0.22	4.57	15.09	0.04	1	7.33	0.77
LOXHD1	ii	Hearing	2,236	96.6	0.11	0.24	5.77	0.03	1	2.20	0.09
MYO3A	ii	Hearing	1,697	96.4	0.18	0.88	10.08	0.02	1	5.88	0.66
MYO6	ii	Hearing	1,215	96	0.05	0.13	4.03	0.02	1	1.00	<0.01
OTOF	ii	Hearing	1,401	70.1	0.05	0.14	7.75	0.02	1	1.00	<0.01
PGAP1	ii	Hearing	750	98.6	0.30	0.90	5.30	0.12	1	3.31	<0.01
ROR1	ii	Hearing	815	91	0.01	0.21	7.12	0.01	1	1.00	<0.01
SCRIB	i	Hearing	656	94.3	0.06	1.31	9.68	0.02	1	4.73	0.39
TBL1X	ii	Hearing	514	98.3	0.03	0.42	5.59	0.02	1	1.00	<0.01
TMC2	ii	Hearing	903	97.2	0.14	0.65	16.89	0.04	1	1.00	<0.01
TMPRSS3	i	Hearing	472	99	0.12	1.30	8.67	0.05	1	4.69	0.41
GPER1	i	Feather kerat.	357	100	0.03	1.28	8.19	0.01	1	1.00	<0.01
KRT5	ii	Feather kerat.	768	60.5	0.05	0.29	6.15	0.02	1	9.26	0.07
TCHP	iii	Feather kerat.	230	78.8	0.35	0.12	0.99	0.10	1	87.68	3.94
CPT1A	i	Circadian rhythm	742	96.4	0.09	2.53	19.58	0.03	1	9.58	0.60
CRY1	ii	circadian rhythm	457	98.9	0.05	0.53	5.69	0.02	1	3.23	−0.78
OPN4-1	ii	Circadian rhythm	482	82.4	0.22	0.64	4.02	0.08	1	1.58	<0.01
SLC6A4	ii	Circadian rhythm	660	98.4	0.08	0.50	6.23	0.04	1	4.02	0.19
STAR	ii	Circadian rhythm	124	42.2	0.05	0.61	4.82	0.04	1	1.00	<0.01

Note.—Genes are classified by functional category (vision, hearing, feather keratin, and circadian rhythm) and sorted alphabetically. List refers to the significant *ω* test categories, whereby list i includes genes with a signal of strong positive selection (*ω*_1_ > 1, branch model), list ii includes genes with a signal of weak positive or relaxed purifying selection (*ω*_0_ < *ω*_1_ < 1, branch model), and list iii includes genes with a signal of site-specific positive selection in the foreground branch (*ω*_2_ > 1, branch-site model).

Twenty-one candidate genes related to vision, ten to hearing, two to feather keratin, and five to circadian rhythm showed a higher *ω* value on the ancestral branch of the owls compared with the background (branch model; table 1, lists i and ii). Three candidate genes had a significant signal of positive selection at specific sites on the ancestral branch of the owls (branch-site model; table 1, list iii): one from the feather keratin category, and the other two from the visual system.

## Discussion

### Genome-Wide Significant Selection on Single Genes in the Owl Ancestor

Our study detected only 22 single genes with genome-wide significant signals of selection at the origin of the owls. The relatively low number of genes is expected because correction for multiple testing is strong in genome analyses and hence only the strongest single signals will pass as significant. These 22 genes encode mostly components of the membrane and are functionally associated with sensory perception (vision and sound), DNA condensation, and lipid metabolism. A formal functional enrichment analysis identified a single significant GO term “detection of stimulus involved in sensory perception” which contains the genes *TMC2*, *PCDH15*, *PPEF2*, and *CACNA2D4*. The first two genes play a role in auditory perception and the latter three in visual perception (NCBI gene db, GeneCards, and AmiGO2). *TMC2* is involved in mechanotransduction in cochlear hair cells of the inner ear, and *PCDH15* participates in the maintenance of normal retinal and cochlear function (GeneCards). *PPEF2* is expressed specifically in photoreceptors and the pineal gland and participates in phototransduction (GeneCards). The protein encoded by *CACNA2D4* plays an important role in the normal functioning of the retina and cardiac tissue because it is involved in transmembrane transport of calcium (GeneCards). The description of the function and related diseases in humans for all 22 genome-wide significant genes is provided in supplementary table S3 in file 2, Supplementary Material online.

### Genome-Wide Functional Overrepresentation of Genes with Nominal Selection Signals in the Owl Ancestor

Further analysis of all nominal significant signals (irrespective of their genome-wide significance and potentially comprising weaker selection signals) for enrichment of functionally related gene sets is recommended, because it can be informative if co-selection of functions or pathways is suspected (Mooney et al. 2014; Mueller et al. 2020). The major functional groups consistently found among the different tests were related to the processes of sensory perception (vision and hearing) and chromosome conformation.

#### Functional Overrepresentation Related to Vision

We found a strong and consistent enrichment of genes related to functions in photoreceptors among genes with an accelerated substitution rate in the origin of the owls (lists i and ii). Several of these genes are relevant for light perception, the first steps in phototransduction, dim-light vision, or the development and maintenance of the retina. Besides being part of the overrepresented visual-related functional groups, three of these genes are also genome-wide significant (*CACNA2D4*, *PCDH15*, and *PPEF2*), and one gene has an owl-specific signal of positive selection (*RP1*). The gene network related to functions in the plasma membrane (list ii, fig. 3) is highly connected to the network of photoreceptor functions (as shown by the many shared genes, fig. 3). This is probably because sensory perception depends on the transduction of the stimuli through reaction cascades on the plasma membrane of the photoreceptors.

The overrepresented functional group linked to photoreceptors comprises ten genes with evidence for strong positive selection (*ω*_foreground_ > 1). Three of these genes have also been identified in previous studies on raptors (*CNGA1*, *SAG*, and *SLC24A1*), are expressed in rods, and play a role in phototransduction and recovery of the rod photoreceptors (Wu et al. 2016)*.* The other seven genes with evidence for strong positive selection (*FAM161A*, *GUCA1C*, *LCA5*, *PPEF2*, *PRPH2*, *RPGRIP1L*, and *SPTBN5*) have not been described before as genes that may have played a role in the early diversification of the owls. Interestingly, Wu et al. (2016) did not find the gene *GUCA1C* in the transcriptome of owls. This gene encodes for a cone-specific protein that participates in photoresponse recovery and the authors suggested that this gene might have been lost or has become a nonfunctional pseudo-gene in the Strigidae. However, our results indicate that this gene is present in owls and has evolved under positive selection in the ancestral branch.

The overrepresented functional group linked to sensory perception includes 50 genes that evolved faster in the owl ancestor (*ω*_background_  <  *ω*_foreground_ < 1). We found confirmatory evidence that four of these genes (*CNGB1*, *ABCA4*, *PCDH15*, and *BEST1*) have evolved faster in the owl ancestor, as reported in previous studies (Wu et al. 2016; Cho et al. 2019). The gene *RP1* is also present in the functional network of genes showing positive selection on specific sites (list iii) and links to a function for microtubules, which might be associated with the development and maintenance of photoreceptors.

#### Functional Overrepresentation Related to Hearing

Several species that are adapted to darkness or dim-light conditions have enhanced hearing or olfaction capabilities, complementing the visual cues by auditory or olfactory information. In birds, the kiwi and the barn owl are well-studied cases. The kiwis are the only nocturnal ratite relying more on olfaction than vision for foraging and this group has evolved an extended repertoire of odorant receptors (Le Duc et al. 2015). Barn owls possess acute hearing and an ability to localize their prey in darkness (Payne 1971). They have several special traits that improve their hearing, such as a facial disk, asymmetrical position of the ears, and resistance to hearing loss by aging (Krumm et al. 2017).

The GO term “sensory perception of sound” is overrepresented among genes with signals of weak positive or relaxed purifying selection in the owl ancestor. Considering the well-developed auditory system of the owls, it seems likely that the elevated *ω* values reflect positive selection either for a short period or with low intensity in the owl ancestor. The ten genes associated with this GO term are *ATP8B1*, *LOXHD1*, *MYO3A*, *MYO6*, *OTOF*, *PCDH15*, *PGAP1*, *ROR1*, *TBL1X*, and *TMC2*. From these genes, *PCDH15* and *TMC2* were also genome-wide significant (see above) and are described in supplementary table S3 in file 2, Supplementary Material online. The other genes are involved in inner ear receptor cell development and nerve formation or related to the cytoskeleton and may thus function in mechanotransduction of sound stimuli (NCBI gene db, GeneCards, and AmiGO2). Mutations in *LOXHD1*, *OTOF*, *PCDH15*, *TBL1X*, and *TMC2* have been associated with hereditary disorders of balance, deafness or hearing loss in humans (NCBI gene db and GeneCards).

#### Overrepresentation in Other Functional Categories

We found consistent evidence that 32 genes (lists i–iii) related to DNA conformation change, chromosome condensation, and chromatid segregation have an accelerated substitution rate in the origin of the owls. From these genes, *ATRX*, *SMC2*, and *SMC5* had also genome-wide significant selection signals, and the latter two had nominal significant selection signals from both models (i.e., are in lists i and iii). This group of genes suggests that owls might have evolved a special type of DNA packaging in the retina, similar to what has been found in the rods of nocturnal mice and primates (Solovei et al. 2009). Nocturnal mammals show an unusual radially inverted pattern of hetero- and euchromatin in the nuclei of the rod photoreceptor cells, which acts as a collecting lens channeling the light efficiently toward the light-sensing outer segments, thereby increasing light availability in the deep layers of the retina (Solovei et al. 2009; Joffe et al. 2014; Tan et al. 2019).

The genes with a positive selection signal at specific sites (branch-site model) are enriched in functional categories related to microtubules, including “mitotic nuclear division” and “sperm flagellum” (fig. 4). Of note, microtubules also play an important role in the visual signal transduction cascade of the photoreceptor sensory cilium. The functional overrepresentation associated with the “sperm flagellum” is somewhat unexpected, because owls seem to be strictly genetically monogamous (Lawless et al. 1997; Müller et al. 2001; Rodriguez-Martínez et al. 2014). Genetic monogamy does not promote sperm competition and selection on sperm morphology (Lifjeld et al. 2010; Rowe et al. 2015; Carballo et al. 2019). However, the results from the branch-site model should be interpreted cautiously due to the potential influence of CMDs (Venkat et al. 2018).

### Selection in A Priori Defined Candidate Genes Related to the Nocturnal Predatory Lifestyle of Owls

#### Candidate Genes Related to Vision

The gene *RP1* is the only candidate gene that was significant in both the branch and the branch-site model (lists ii and iii). Furthermore, *RP1* was also significant according to the model aBSREL, indicating a signal of positive selection that is specific for the ancestral branch of the owls. *RP1* encodes a retinal-specific protein related to photosensitivity and the outer segment morphogenesis of rod photoreceptors and is essential for nocturnal vision. *RP1* is also a microtubule-associated protein, required for correct stacking of the outer segment disks.

Our finding that the genes *RGS9*, *BEST1*, *RRH*, *RDH8*, *RPE65*, *PDE6B*, and *ALCAM* evolved faster in the ancestral branch of owls than in the background branches, partially confirm previous results for nocturnal birds and raptors (Wu et al. 2016; Cho et al. 2019; Zhou et al. 2019). These genes are functionally related to visual perception, photoreceptor activity, phototransduction cascades, regeneration of visual pigments, and retina development, and some of them have been linked to genetic diseases related to vision in humans. Our MSA also confirmed the two owl-specific missense mutations in *ALCAM* first reported by Zhou et al. (2019, fig. 3d), which presumably change the charge of a relevant region of the protein surface from neutral to negative.

We found evidence for relaxed purifying selection in the opsin gene *OPN1MSW* on the ancestral branch of the owls, which fits the described pseudogenization of this gene in tytonids (Borges et al. 2015; Wu et al. 2016; Hanna et al. 2017). The opsin genes *OPN1LW* and *SWS2* were not found in the burrowing owl assembly. This is likely an assembly or annotation error because previous studies showed that owls have retained these two cone opsin genes (Borges et al. 2015; Wu et al. 2016; Hanna et al. 2017). Moreover, Wu et al. 2016 found signals of positive selection for both genes at the ancestral branch of owls and suggested that this might be adaptive for crepuscularity. The opsin genes *RHO* and *RGR* were detected and tested, but the *ω* tests were not significant.

#### Candidate Genes Related to Hearing

We found evidence for an accelerated substitution rate at the ancestral branch of the owls in the hearing-related candidate genes *LOXHD1*, *MYO3A*, *MYO6*, *OTOF*, *PGAP1*, *ROR1*, *SCRIB*, *TBL1X*, *TMPRSS3*, and *TMC2*. *SCRIB*, *TMPRSS3*, and *TMC2* showed the strongest signal of positive selection, the first two in terms of the *ω* value (table 1) and the third in terms of *P* value. *SCRIB* is involved in different aspects of polarized cell differentiation, regulating epithelial and neuronal morphogenesis. *TMPRSS3* is expressed in the fetal cochlea, probably participating in the development and maintenance of the inner ear. Mutations in *TMPRSS3* are associated with congenital deafness in humans (NCBI gene db, GeneCards, and AmiGO2).

#### Candidate Genes Related to Circadian Rhythm

The genes involved in the molecular mechanism behind the circadian rhythm, for example, those coding for nonvisual photopigments, are mostly conserved across mammals and birds (Yoshimura et al. 2000; Bhadra et al. 2017). Our results show an accelerated substitution rate at the ancestral branch of the owls in five candidate genes related to circadian rhythm and sleep: *OPN4-1*, *CRY1*, *CPT1A*, *STAR*, and *SLC6A4*. Our finding of *OPN4-1*, a nonvisual opsin, as a candidate gene is consistent with previous studies on nocturnal birds (Borges et al. 2015; Le Duc et al. 2015; Cho et al. 2019). *CRY1* is a central component of the circadian clock (Griffin et al. 1999). *CPT1A* encodes a key protein for the mitochondrial oxidation of long-chain fatty acids and is linked to the GO term “circadian rhythm” (GeneCards and AmiGO2) and the “circadian clock” pathway (GeneCards and reactome: reactome.org/PathwayBrowser/#/R-HSA-400253) in humans. The protein encoded by *STAR* plays a role in the regulation of steroid hormone synthesis by mediating the transport of cholesterol through the mitochondrial membrane and is linked to the GO terms “circadian rhythm” and “circadian sleep/wake cycle, REM sleep” in humans (GeneCards and AmiGO2). *SLC6A4* regulates synaptic concentrations of serotonin, indirectly influencing perception and anxiety-related behavior. *SLC6A4* and *CRY1* have been related to sleep disorders in humans (Carskadon et al. 2012; Patke et al. 2017).

Cho et al. (2019) found a burrowing owl-specific amino-acid variant in *SLC51A.* Cho et al. associate this amino-acid variant with the diurnality of this species, because the gene is associated with bile acid transmembrane transporter activity and has an indirect effect on the circadian rhythm. We did not find evidence for selection on *SLC51A*, but we confirmed this variant for the burrowing owl and its congeneric, the little owl, indicating that this pattern might be associated with the *Athene* taxon, but not necessarily with diurnality.

#### Candidate Genes Related to Feather Structure

The feathers of owls have a special noise absorption structure that allows them to fly silently while hunting, and this feature has been studied morphologically and acoustically (Kopania 2016; Sagar et al. 2017; Weger and Wagner 2017). However, the genetic correlates of this adaptation in owls remain unclear.

Here, we present evidence for positive selection in the ancestral branch of the owls for three candidate genes related to feather production: *GPER1*, *TCHP*, and *KRT5*. *GPER1* and *TCHP* are related to keratin filament development and production. The gene *KRT5* belongs to the keratin gene family; it is co-expressed during differentiation of simple and stratified epithelial tissues and is important for keratinization, cornification, and epidermis development (GeneCards and AmiGO2).

## Conclusions

We conducted a genome-wide comparative analysis focusing on the early history of Strigiformes. Our study suggests novel candidate genes whose role in the evolution of owls can be further explored. Our study also contributes the raw genome sequencing data of eight owl species (NCBI BioProject PRJNA592858).

Our results support that owls—similar to other nocturnal birds—early on evolved sensory adaptations that allowed them to cope with dim light. In particular, phototransduction in the rods, enhanced motion detection and retina repair, but also acoustic perception seem to be important for the owls. We also found evidence for functional overrepresentation associated with chromosome packaging. This suggests a role of chromatin packaging for enhanced light channeling in photoreceptor cells as a target of adaptation in the owl ancestor. The information-driven approach also supports the idea that genes involved in feather development and circadian rhythm have evolved under positive selection in the ancestral branch of the owls.

In agreement with the diurnal ancestry of raptorial landbirds, our results show the accumulation of genetic changes in several genes functionally associated with nocturnal hunting, indicating the independent adaptive history of owls as nocturnal birds of prey.

## Supplementary Material

evaa166_Supplementary_Data

## Notes

Data deposition: Raw sequences have been deposited at the short-read archive (SRA) of the NCBI db under the BioProject PRJNA592858.
