# Supplementary material for: Genomic Evidence for Sensorial Adaptations to a Nocturnal Predatory Lifestyle in Owls
Source: Genome Biol Evol. 2020 Aug 8;12(10):1895–908. doi: 10.1093/gbe/evaa166 (PMC7566403; doi:10.1093/gbe/evaa166)
Supplement: evaa166_Supplementary_Data [file evaa166_supplementary_data.zip › Espíndola-Hernández_etal_2019_SupplementaryFile_1_R2.pdf]

Supplementary File 1

Genomic evidence for sensorial adaptations to a nocturnal predatory lifestyle in owls

Pamela Espíndola-Hernández[1*], Jakob C. Mueller[1], Martina Carrete[2], Stefan Boerno[3], Bart Kempenaers[1]

[1] Department of Behavioural Ecology and Evolutionary Genetics, Max Planck Institute for Ornithology, Seewiesen, Germany
[2] Department of Physical, Chemical and Natural Systems, Universidad Pablo de Olavide, Sevilla, Spain
[3] Sequencing Core Facility, Max Planck Institute for Molecular Genetics, Berlin, Germany
[*] Corresponding author: pespindola@orn.mpg.de

1 Extended Materials and Methods

Table S1. Samples and pairwise sequence alignment information.
Table S2. Candidate gene list by functional categories.
Figure S1. Workflow to test selection on the ancestral branch of owls.

1.1 Commands
Protocol a: Read mapping to reference
Protocol b: Genome-scale sequence mapping to reference
Multi-species Codon Alignment
Quality assessment of multispecies alignments
Selection test
Newick format of the unrooted species tree
Overrepresentation analyses

2 Extended Results

BUSCO summary
Figure S2. **a, b.** Proportion of CMDs and the Branch-Site Model
Table S3. (in Supplementary File 2*) Description of genes with genome-wide significant ω tests.
Table S4. (in Supplementary File 2*) Significant results from the branch model.
Table S5. (in Supplementary File 2*) Significant results from the branch-site model.
Table S6. (in Supplementary File 2*) Significant results from the aBSREL model if two rate classes were estimated.
Table S7. Overrepresented functional GO-groups of gene list i, ii, iii by ClueGO.
Table S8. Overrepresented functional GO-terms of gene list i, ii, iii by ClueGO.
Table S9. Overrepresented functional GO-terms of gene list i, ii, iii with family-wise error rate (FWER) < 0.05 identified by GOfunR based on 10000 permutations.
Table S10. (in Supplementary File 2*) Results of the ω test for all candidate genes, including non-significant results.

1 Extended Materials and Methods

Table S1. Samples and pairwise sequence alignment information. The assembly of *Athene cunicularia* (Burrowing owl, assembly athCun1) was used as a reference genome with a total length of 1,157,069,517 bp, a codon sequence length of 26,451,662 bp, and an N50 value of 42,147,404 bp. The table lists basic information for each sample or genome data and pairwise alignment characteristics of each species (gaps and percentage of the reference genome covered, where "N" represents a site with zero coverage in relation to the reference. Separate information is given for the genome and for the coding region (CDS).

							All genome				CDS	
Scientific name	Common name	Data/sample origin	Publication	GenBank assembly accession	Sex	Average read Depth	"N" count (bp)	Gaps count	% covered	% of "N" in the alignment	% Covered	% of "N" in the alignment
Athene noctua	Little owl	Blood in ethanol, UMH, Spain	This study	PRJNA592858[1]	male	17.64	25,094,316	1,186,824	97.83	2.17	99.21	0.79
Surnia ulula	Northern hawk-owl	Blood in ethanol, Zoo Zurich, Switzerland	This study	PRJNA592858[1]	male	26.19	34,668,645	1,459,917	97.01	2.99	99.04	0.96
Bubo scandiacus	Snowy owl	Liver in buffer, Zoo Antwerp, Belgium	This study	PRJNA592858[1]	female	26.80	48,146,487	1,732,063	95.85	4.15	98.63	1.37
Bubo bubo	Eurasian eagle-owl	Blood in ethanol, UMH, Spain	This study	PRJNA592858[1]	male	24.95	48,541,302	1,844,811	95.81	4.19	98.58	1.42
Strix uralensis	Ural owl	Blood in ethanol, Zoo Zurich	This study	PRJNA592858[1]	female	21.84	53404,103	1,839,472	95.65	4.35	98.46	1.54
Strix nebulosa	Great grey owl	Blood in ethanol, Zoo Zurich	This study	PRJNA592858[1]	female	18.84	52,167,639	1,950,101	95.50	4.50	98.40	1.60
Asio otus	Long-eared owl	Blood in ethanol, UMH, Spain	This study	PRJNA592858[1]	male	15.21	50,328,877	2,457,902	95.65	4.35	98.27	1.73
Asio flammeus	Short-eared owl	Blood in ethanol, UMH, Spain	This study	PRJNA592858[1]	male	15.30	51,462,491	2,358,876	95.55	4.45	97.51	2.49
Strix occidentalis	Spotted owl	NCBI	Hanna et al. 2017	GCA_002372975.1	female		65,347,625	2,755,189	94.35	5.65	97.41	2.59
Tyto alba	Barn owl	NCBI	Zhang et al. 2014	GCA_000687205.1	female		113,335,374	3,771,159	90.20	9.80	96.85	3.15
Haliaeetus leucocephalus	Bald eagle	NCBI	Zhang et al. 2014	GCA_000737465.1	male		132,070,523	4,106,734	88.59	11.41	94.43	5.57
Falco peregrinus	Peregrine falcon	NCBI	Zhan et al. 2013	GCA_000337955.1	male		150,796,022	5,811,744	86.97	13.03	94.14	5.86
Leptosomus discolor	Cuckoo roller	NCBI	Zhang et al. 2014	GCA_000691785.1	male		156,539,425	5,197,363	86.47	13.53	92.06	7.94
Gallus gallus (Ggallus5)	Red junglefowl	NCBI	International Chicken Genome Consortium 2015	GCA_000002315.3	female		469,639,409	6,240,954	59.41	40.59	91.78	8.22
Cathartes aura	Turkey vulture	NCBI	Zhang et al. 2014	GCA_000699945.1	female		132,420,611	3,549,849	88.56	11.44	90.55	9.45
Picoides pubescens	Downy woodpecker	NCBI	Zhang et al. 2014	GCA_000699005.1	female		433,822,859	5,781,688	62.51	37.49	88.86	11.14
Colius striatus	Speckled mousebird	NCBI	Zhang et al. 2014	GCA_000690715.1	male		272,689,826	6,596,761	76.43	23.57	88.28	11.72
Taeniopygia guttata (taeGut2)	Zebra finch	NCBI	Warren et al. 2010	GCA_000151805.2	male		362,730,059	7,279,320	68.65	31.35	88.20	11.80
Apaloderma vittatum	Bar-tailed trogon	NCBI	Zhang et al. 2014	GCA_000703405.1	male		253,133,073	5,972,504	78.12	21.88	87.95	12.05

Raw sequences have been submitted to the short read archive (SRA) of the NCBI db under BioProject PRJNA592858.

Hanna ZR et al. 2017. Northern spotted owl (*Strix occidentalis caurina*) genome: Divergence with the barred owl (*Strix varia*) and characterization of light-associated genes. Genome Biol. Evol. 9:2522–2545. doi: 10.1093/gbe/evx158.

Warren WC et al. 2010. The genome of a songbird. Nature. 464:757–762. doi: 10.1038/nature08819.

Zhan X et al. 2013. Peregrine and saker falcon genome sequences provide insights into evolution of a predatory lifestyle. Nat Genet. 45(5):563-6

Zhang G et al. 2014. Comparative genomics reveals insights into avian genome evolution and adaptation. Science. 346:1311–1320. doi: 10.1126/science.1251385.

Table S2. Candidate gene list by functional categories**.**

	Gene	candidate category		Gene	candidate category
1	AANAT	circadian rhythm	128	SCRIB	hearing
2	ABCC9	circadian rhythm	129	SEC24B	hearing
3	ADA	circadian rhythm	130	SLC26A5	hearing
4	ADORA2A	circadian rhythm	131	SLC9A3R1	hearing
5	AHCY	circadian rhythm	132	SOBP	hearing
6	ARNTL	circadian rhythm	133	SOD1	hearing
7	ARNTL2	circadian rhythm	134	SPRY2	hearing
8	BHLHE40	circadian rhythm	135	STRC	hearing
9	BTBD9	circadian rhythm	136	TBL1X	hearing
10	CACNA1C	circadian rhythm	137	TECTA	hearing
11	CACNA1I	circadian rhythm	138	TJP1	hearing
12	CIPC	circadian rhythm	139	TMC1	hearing
13	CLOCK	circadian rhythm	140	TMC2	hearing
14	CPT1A	circadian rhythm	141	TMIE	hearing
15	CREB1	circadian rhythm	142	TMPRSS3	hearing
16	CRHR1	circadian rhythm	143	TRIOBP	hearing
17	CRY1	circadian rhythm	144	TUB	hearing
18	CSNK1D	circadian rhythm	145	USH1C	hearing
19	CSNK2A2	circadian rhythm	146	USH1G	hearing
20	DIO2	circadian rhythm	147	USH2A	hearing
21	DLAT	circadian rhythm	148	WDPCP	hearing
22	FBXL3	circadian rhythm	149	WHRN	hearing
23	FOS	circadian rhythm	150	ABCA4	vision
24	FSHB	circadian rhythm	151	ALCAM	vision
25	GHRL	circadian rhythm	152	ARR3	vision
26	GNB3	circadian rhythm	153	ATP8A2	vision
27	GRIA3	circadian rhythm	154	ATP8B1	vision
28	GRIN2A	circadian rhythm	155	BBS4	vision
29	HCRTR2	circadian rhythm	156	BEST1	vision
30	HDAC3	circadian rhythm	157	BHLHE23	vision
31	HTR2A	circadian rhythm	158	CACNA2D4	vision
32	HTR7	circadian rhythm	159	CACNB2	vision
33	ID2	circadian rhythm	160	CACNB4	vision
34	IL18	circadian rhythm	161	CCDC66	vision
35	MAOA	circadian rhythm	162	CLN5	vision
36	MTNR1A	circadian rhythm	163	CLN6	vision
37	NAMPT	circadian rhythm	164	CLN8	vision
38	NCOR1	circadian rhythm	165	CNGA1	vision
39	NFIL3	circadian rhythm	166	CNGA3	vision
40	NOCT	circadian rhythm	167	CNGB1	vision
41	NPAS2	circadian rhythm	168	CNGB3	vision
42	NPSR1	circadian rhythm	169	COL11A1	vision
43	NR0B2	circadian rhythm	170	CRABP1	vision
44	NR1D2	circadian rhythm	171	CRYBA1	vision
45	NRIP1	circadian rhythm	172	DMD	vision
46	NT5E	circadian rhythm	173	DNAJC19	vision
47	OPN4	circadian rhythm	174	EPAS1	vision
48	OPN4-1	circadian rhythm	175	EPHB2	vision
49	OXTR	circadian rhythm	176	EYS	vision
50	PCSK2	circadian rhythm	177	GABRR2	vision
51	PER2	circadian rhythm	178	GJD2	vision
52	PER3	circadian rhythm	179	GLRA1	vision
53	PPARGC1A	circadian rhythm	180	GLRB	vision
54	PPP1CB	circadian rhythm	181	GNAT1	vision
55	PRKG1	circadian rhythm	182	GNAT2	vision
56	PROK1	circadian rhythm	183	GNB1	vision
57	PROK2	circadian rhythm	184	GNB5	vision
58	RAI1	circadian rhythm	185	GNGT2	vision
59	SLC29A1	circadian rhythm	186	GRK7	vision
60	SLC29A3	circadian rhythm	187	GUCA1A	vision

61	SLC6A4	circadian rhythm	188	GUCA1B	vision
62	SRD5A1	circadian rhythm	189	GUCA1C	vision
63	SRRD	circadian rhythm	190	GUCY2F	vision
64	STAR	circadian rhythm	191	HCN1	vision
65	TEF	circadian rhythm	192	ISL1	vision
66	TH	circadian rhythm	193	KCNA2	vision
67	TRIB1	circadian rhythm	194	LAMC3	vision
68	CSNK1A1	feather keratin	195	LUM	vision
69	EPPK1	feather keratin	196	MYO5A	vision
70	FAM83H	feather keratin	197	MYO7A	vision
71	FBF1	feather keratin	198	NAV2	vision
72	GPER1	feather keratin	199	NOB1	vision
73	KRT14	feather keratin	200	NR2E1	vision
74	KRT5	feather keratin	201	NRP1	vision
75	KRT6A	feather keratin	202	NRP2	vision
76	KRT7	feather keratin	203	NTRK2	vision
77	KRT71	feather keratin	204	NYX	vision
78	KRT75	feather keratin	205	OPA1	vision
79	KRT8	feather keratin	206	OPN1MSW	vision
80	TCHP	feather keratin	207	OPN3	vision
81	ALG10	hearing	208	OPN5	vision
82	ATP6V0A4	hearing	209	OPTC	vision
83	CACNA1D	hearing	210	OPTN	vision
84	CDC14A	hearing	211	PCDH15	vision
85	CDH23	hearing	212	PDCL	vision
86	CDKN1B	hearing	213	PDE5A	vision
87	CEMIP	hearing	214	PDE6B	vision
88	CHD7	hearing	215	PDE6C	vision
89	CHRNA9	hearing	216	PDE6D	vision
90	CLIC5	hearing	217	PDE6G	vision
91	CLRN1	hearing	218	PDE6H	vision
92	COCH	hearing	219	PHOX2B	vision
93	CRYM	hearing	220	PLXNA4	vision
94	DCDC2	hearing	221	PPT1	vision
95	DNER	hearing	222	PRPH2	vision
96	EPYC	hearing	223	RARB	vision
97	EYA1	hearing	224	RBP4	vision
98	EYA4	hearing	225	RDH10	vision
99	FBXO11	hearing	226	RDH8	vision
100	FZD4	hearing	227	REEP6	vision
101	GABRA5	hearing	228	RGR	vision
102	GABRB2	hearing	229	RGS9	vision
103	GABRB3	hearing	230	RGS9BP	vision
104	GPX1	hearing	231	RHO	vision
105	HEXA	hearing	232	RORB	vision
106	HEXB	hearing	233	Rp1	vision
107	HOMER2	hearing	234	RPE65	vision
108	KCNQ4	hearing	235	RPGR	vision
109	KIT	hearing	236	RRH	vision
110	LHFPL5	hearing	237	RS1	vision
111	LOXHD1	hearing	238	SAG	vision
112	LRIG1	hearing	239	SALL1	vision
113	LRIG2	hearing	240	SEMA3A	vision
114	LRP2	hearing	241	SEMA3F	vision
115	MARVELD2	hearing	242	SIX4	vision
116	MKKS	hearing	243	SLC1A3	vision
117	MYO3A	hearing	244	SLC24A1	vision
118	MYO6	hearing	245	SLC24A2	vision
119	NR4A3	hearing	246	SLITRK6	vision
120	OTOF	hearing	247	TFAP2A	vision
121	OTOGL	hearing	248	THY1	vision
122	OTOS	hearing	249	TMEM126A	vision
123	PDZD7	hearing	250	TRPM1	vision

124	PGAP1	hearing	251	TULP1	vision
125	PTPRQ	hearing	252	VSX1	vision
126	RIPOR2	hearing	253	WFS1	vision
127	ROR1	hearing			

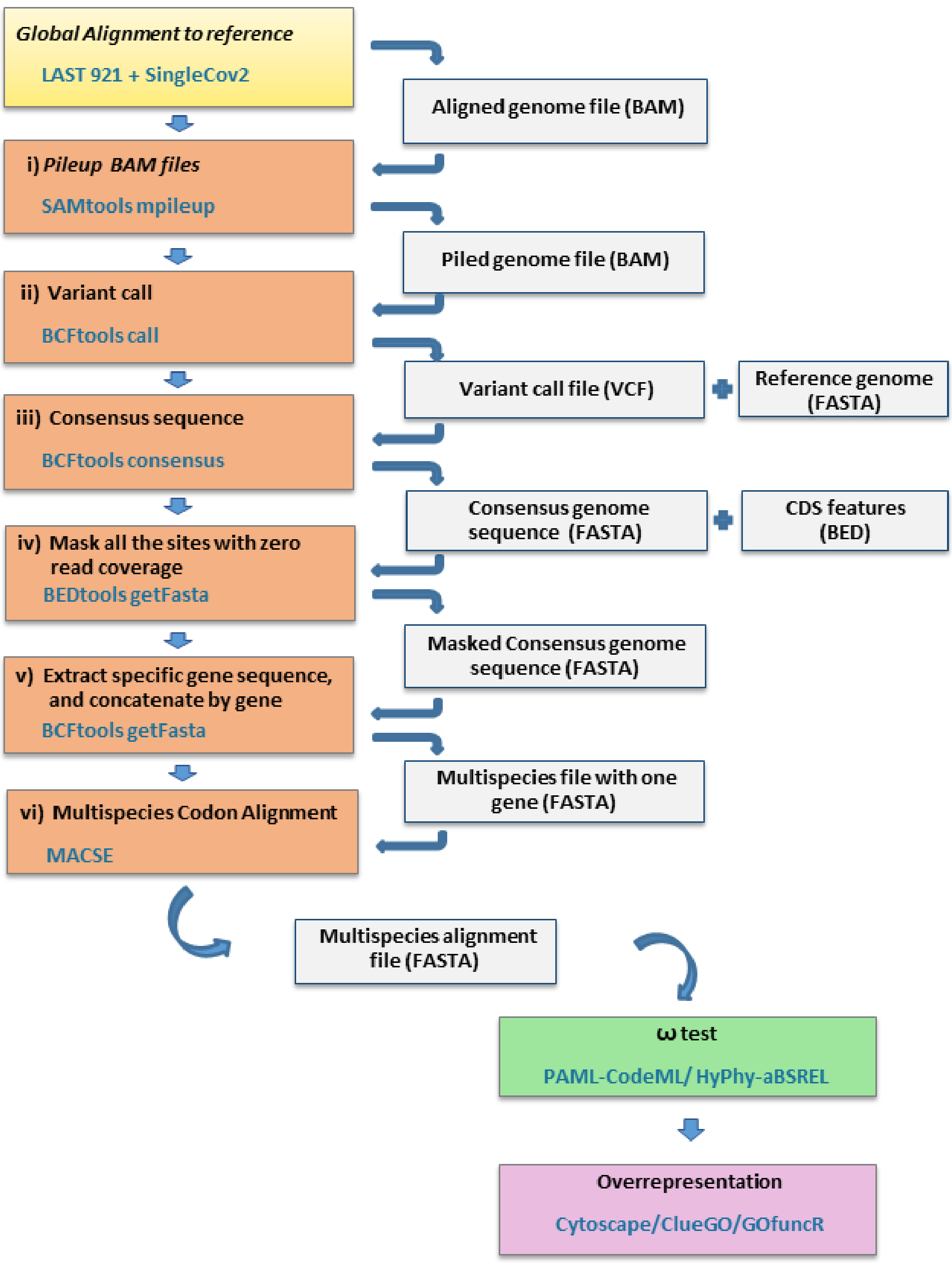

Fig. S1. Workflow to test selection on the ancestral branch of owls. After global genome alignment to the reference genome (yellow), six steps produce the multispecies codon alignment of each gene (orange). Finally, selection tests by ω=dN/dS estimations (green) and overrepresentation analyses (purple) are performed. The names of the software used on each step are given in blue within boxes.

1.1 Commands

The parameter used with each software are detailed below, excluding the infile/outfile.

Protocol a: Read mapping to reference

The reads were mapped against the reference genome using bwa (alignment via Burrows-Wheeler transformation), version: 0.7.17-r1188

bwa[1] mem -M -R

Protocol b: Genome-scale sequence mapping to reference

We aligned species genome assemblies to the reference using LAST v. 921:

lastdb[2] -uMAM8 -cR11
lastal[2] -E0.001 -i3G -m100
SingleCov2[3]
maf-convert[2] sam
samtools[4] view -bS

Multi-species Codon Alignment

i) Piling up the reads or genome sequences in the coding regions
Protocol a:
samtools[4, a] mpileup -u -f athCun1.fa -l CDS.bed -I --output-tags AD,INFO/AD,DP,SP
Protocol b:
samtools [b] mpileup -u -f athCun1.fa -l CDS.bed -I -A --output-tags AD,INFO/AD,DP,SP

ii) Variant calling,
Protocol a:
bcftools[5, a] call -m
protocol b:
bcftools [b] call -m –A

iii) Producing consensus sequences :
bcftools[5] consensus -f

iv) Masking all the sites with zero coverage:
bedtools[6] genomecov bga
bedtools[6] maskfasta

v) Producing one multispecies FASTA file for each gene using BEDtools.
bedtools[6] getfasta

vi) Multispecies codon alignment for each gene using MACSE[7]:
macse -prog alignSequences
fas2phy.R[8]

---

[1]bwa: http://bio-bwa.sourceforge.net/
[2]LAST: http://last.cbrc.jp/
[3]Multiz-tba.012109: https://www.bx.psu.edu/miller_lab/
[4]SAMtools: http://www.htslib.org/doc/samtools.html
[5]BCFtools: http://www.htslib.org/doc/bcftools.html
[6]BEDtools: https://bedtools.readthedocs.io/en/latest/
[7]MACSE: https://bioweb.supagro.inra.fr/macse/
[8]fas2phy, Converts FASTA files into PHYLIP format: https://github.com/fmichonneau/chopper/tree/master/R

Quality assessment of multispecies alignments

Two steps trimming with BMGE (version 1.12) [9]

Step 1: Remove the gaps ignoring entropy:

BMGE -t CODON -h 1 -w 1 -g 0

Step 2: Remove blocks of codons with high entropy:

BMGE -t CODON -m BLOSUM62

Summary statistics of multispecies alignments

AMAS.py summary -f fasta -d dna -c 40

[9] BMGE: https://bioweb.pasteur.fr/packages/pack@BMGE@1.12

Selection tests

We tested for accelerated ω on the ancestral branch of Strigiformes using a maximum-likelihood method implemented in the CodeML program in PAML 4.9h[10] using the following settings in the control files:

Branch model

Null hypothesis (H_0)

model = 0 * models for codons: 0: one ω ratio for all branches, 1: one ω ratio for each branch, 2: 2 or more ω ratio for branches
NSsites = 0 * 0: one estimated ω; 1: Nearly neutral; 2: Positive selection
fix_kappa= 0 * 1: kappa fixed, 0: kappa to be estimated
kappa = 2 * initial or fixed kappa value
fix_omega= 0 * 1: omega or omega_1 fixed, 0: estimate
omega = 1 * initial or fixed omega value
cleandata = 1 * remove sites with ambiguity data (1:yes, 0:no)

Alternative hypothesis (H_1)

model = 2
NSsites = 0
fix_kappa = 0
kappa = 2
fix_omega = 0
omega = 1
cleandata = 1 * remove sites with ambiguity data (1:yes, 0:no)

Branch-Site

Null hypothesis (H_0)

model = 2 * models for codons: 0: one ω ratio for all branches, 1: one ω ratio for each branch, 2: 2 or more ω ratio for branches
NSsites = 2 * 0: one estimated ω; 1: NearlyNeutral; 2: Positive selection
fix_kappa = 0
kappa = 2
fix_omega = 1
omega = 1
cleandata = 1 * remove sites with ambiguity data (1:yes, 0:no)

Alternative hypothesis (H_1)

model = 2
NSsites = 2
fix_kappa = 0
kappa = 2
fix_omega = 0
omega = 1.3
cleandata = 1 * remove sites with ambiguity data (1:yes, 0:no)

[10] PAML: http://abacus.gene.ucl.ac.uk/software/paml.html

Newick format of the unrooted species tree used for selection test in CodeML

(((Falco peregrinus,Taeniopygia guttata),((((Picoides pubescens Apaloderma vittatum), Leptosomus discolor), Colius striatus),(((((Bubo bubo,Bubo scandiacus),((Strix uralensis,Strix nebulosa),Strix occidentalis)),(Asio otus,Asio flammeus)),((Athene cunicularia,Athene noctua),Surnia ulula)),Tyto alba)#1)),(Cathartes aura,Haliaeetus leucocephalus),Gallus gallus);

HyPhy/HYPHYMPI[11]

- > with *a priori* specified foreground

HYPHYMPI absrel --branches Foreground

- > without *a priori* specified foreground

HYPHYMPI absrel

Overrepresentation analyses

ClueGO[12] v2.5.4 plug-in for Cytoscape[13]

Statistical Test = Enrichment (Right-sided hypergeometric test)
Correction Method for multiple testing = Bonferroni
GO Fusion = true
GO Group = true
Kappa Score Threshold = 0.4
Min GO Level = 3, Max GO Level = 8
Over View Term = SmallestPValue
Group By Kappa Statistics = true

GOfuncR[14]

results = go_enrich(infile, annotations=CustomAnnot_AthCun, n_randset=10000)

* infile is the list of genes with significant ω values, *i.e.*: list i, ii, iii or the list of genome-wide significant genes.

---

[11]HyPhy: https://stevenweaver.github.io/hyphy-site/methods/selection-methods/
[12]ClueGO: http://apps.cytoscape.org/apps/cluego
[13]Cytoscape: https://cytoscape.org/
[14]GOfuncR: https://bioconductor.org/packages/release/bioc/vignettes/GOfuncR/inst/doc/GOfuncR.html

2 Extended Results

BUSCO summary

BUSCO version is: 4.0.6
The lineage dataset is: aves_odb10 (Creation date: 2019-11-20)
Summarized benchmarking in BUSCO notation for file athCun1.fa
BUSCO was run in mode: genome

C:94.8%[S:94.6%,D:0.2%],F:1.6%,M:3.6%,n:8338

7905	Complete BUSCOs (C)
7887	Complete and single-copy BUSCOs (S)
18	Complete and duplicated BUSCOs (D)
131	Fragmented BUSCOs (F)
302	Missing BUSCOs (M)
8338	Total BUSCO groups searched

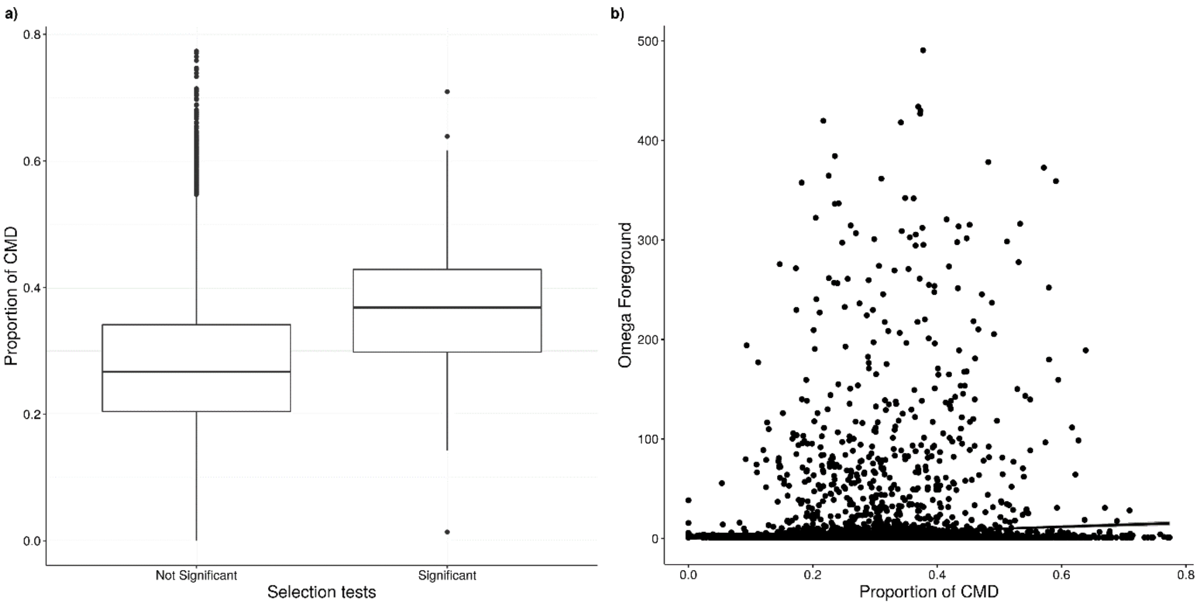

Fig. S2. Proportion of CMDs and the Branch-Site Model. a) Box-plots comparing the proportion of CMDs among the significant tests and the non-significant tests of the Branch-Site model of CodeML. b) Scatter plot of foreground ω values of the alternative hypothesis in relation to the proportion of CMDs for all genes.

Table S7. Overrepresented functional GO-groups of gene list i,ii,iii by ClueGO.

Group	Function by leading GO term	Genes in the functional group	Group p-value	Bonferroni corrected group p-Value
**List i: Genes evolving under strong positive selection on the foreground (with ω0 ≤ 1 < ω1). N* = 199 genes**				
3	photoreceptor cell cilium	CNGA1, FAM161A, GUCA1C, LCA5, PPEF2, PRPH2, RPGRIP1L, SAG, SLC24A1, SPTBN5	<0.001	<0.001
1	positive regulation of chromosome segregation	DLGAP5, SLF1, SMC5, SMC6	<0.001	<0.001
4	chromosome condensation	CENPP, CENPT, CHD1, ERCC8, GPER1, M1AP, NCAPD2, NCAPG, NUSAP1, SETX, SMC2	<0.001	<0.001
2	nuclear chromosome segregation	DLGAP5, M1AP, MEIOC, NCAPD2, NCAPG, NUSAP1, SGO2, SLF1, SMC2, SMC5	0.001	0.003
**List ii: Genes evolving under relaxed purifying or weak positive selection on the foreground (with ω0 < ω1 < 1), N* = 287 genes**				
7	sensory perception	ABCA4, ABLIM1, ACPP, ADCY5, ANO2, ARR3, ATP8B1, BEST1, CACNA2D4, CEP290, CHRNA4, CNGB1, CRY1, DDHD2, DOP1B, GABRA6, GABRR2, GRIN2C, GRK7, GUCA1B, GUCY2F, IMPG1, IMPG2, KCNK2, LOXHD1, MMP24, MYO3A, MYO6, OPN1MSW, OTOF, PCDH15, PGAP1, PLA2G6, PRDM1, PRDX1, RD3, RGS9, RIMS2, ROR1, RP1, RP1L1, RPE65, SCNN1B, SHISA9, SLC6A4, TAS1R3, TBL1X, TMC2, UNC13B, ZMPSTE24	<0.001	<0.001
6	plasma membrane bounded cell projection	ABCA4, ABLIM1, ACPP, ADCY5, ADCY9, ANO2, APPL2, ARHGEF4, ARR3, ATP6V1A, ATP8B1, CDK6, CEL, CEP170, CEP290, CHRNA4, CLIP1, CNGB1, CRY1, CTSZ, DAGLA, DNAH3, DNAH8, DNAH9, DNM1, DTNA, DYSF, ENO2, GABRA6, GRK7, GUCA1B, GUCY2F, HSPA2, ITPR1, KCNK2, KIF4A, KLHL24, LAMA2, LAMP5, LOXHD1, LRP4, LRP8, MARK1, MYO3A, MYO6, NEK8, OPN1MSW, OTOF, PCDH15, PDLIM5, PTPRS, RGS9, RIMS2, ROR1, RP1, RP1L1, RPH3A, RTTN, SBF2, SHISA9, SLC16A3, SLC40A1, SLC6A4, STAR, SYNE2, SYT11, TMC2, UNC13B, UNC13C, UNC5A, WRN	<0.001	<0.001
5	myosin complex	ACTR10, CGNL1, DNAH3, DNAH8, KIF15, KIF4A, LRP8, MYBPC1, MYH11, MYH15, MYL1, MYO3A, MYO6, RP1, TPR	<0.001	<0.001
4	sensory perception of sound	ATP8B1, LOXHD1, MYO3A, MYO6, OTOF, PCDH15, PGAP1, ROR1, TBL1X, TMC2	<0.001	0.001
1	DNA conformation change	ASH1L, ATRX, CHAF1A, FANCM, HP1BP3, MIS18BP1, MNAT1, NCAPH, RAD54B, SRPK1, TOP2A, TPR, WRN	<0.001	0.001
2	supramolecular fiber	AKAP13, ASPM, CCT6A, CDC27, CEP170, CKAP5, CLIP1, COL3A1, COL5A1, DNAH3, DNAH8, DNAH9, DNM1, DYSF, KIF4A, KRT5, MYBPC1, MYH11, MYH15, MYL1, MYO3A, MYO6, NEB, OBSL1, RP1, RP1L1, SYNE2	<0.001	0.001
3	transmembrane transporter complex	ANO2, ATP12A, BEST1, CACNA2D4, CHRNA4, CNGB1, GABRA6, GABRR2, GRIN2C, HSPA2, KCNG4, KCNK2, SCNN1B, SHISA9	<0.001	0.001
**List iii: Genes evolving under positive selection on specific sites of the foreground branch, N* = 123 genes**				
2	tubulin binding	CLIP1, GAS8, INO80, KIF20B, KIF4A, MAP7D3, NUSAP1, RP1, SAXO1	<0.001	0.001
3	sperm flagellum	DNAH1, GAS8, SAXO1, TEKT4	0.002	0.007
1	microtubule bundle formation	CLIP1, DNAH1, GAS8, RP1	0.003	0.011
4	mitotic nuclear division	INO80, KIF20B, KIF4A, MTBP, NUSAP1, SLF1, SMC2, SMC5	0.003	0.012

* Number of genes after all filtering steps.
A complete list of the GO-terms identified for each list of genes and their statistical support are in Table S8.

Table S8. Overrepresented functional GO-terms of gene list i,ii,iii by ClueGO.

GO ID	GO Term	Associated genes	Ontology category	Associated GO group	% associated genes	Nº associated genes	Raw p-Value	Bonferroni corrected p-Value
**List i: Genes evolving under strong positive selection on the foreground (with ω0 ≤ 1 < ω1). N* = 199 genes**								
GO:0097733	photoreceptor cell cilium	CNGA1, FAM161A, GUCA1C, LCA5, PPEF2, PRPH2, RPGRIP1L, SAG, SPTBN5	CC	3	10.59	9	<0.001	<0.001
GO:0030261	chromosome condensation	GPER1, NCAPD2, NCAPG, NUSAP1, SMC2	BP	4	22.73	5	<0.001	0.001
GO:0071103	DNA conformation change	CENPP, CENPT, CHD1, ERCC8, GPER1, M1AP, NCAPD2, NCAPG, NUSAP1, SETX, SMC2	BP	4	6.47	11	<0.001	0.002
GO:0006323	DNA packaging	CENPP, CENPT, GPER1, M1AP, NCAPD2, NCAPG, NUSAP1, SMC2	BP	4	8.70	8	<0.001	0.003
GO:0000796	condensin complex	NCAPD2, NCAPG, SMC2	CC	4	42.86	3	<0.001	0.005
GO:0051984	positive regulation of chromosome segregation	DLGAP5, SLF1, SMC5, SMC6	BP	1	17.39	4	<0.001	0.015
KEGG:04744	Phototransduction	CNGA1, GUCA1C, SAG, SLC24A1	KEGG	3	17.39	4	<0.001	0.015
GO:0001750	photoreceptor outer segment	CNGA1, GUCA1C, PPEF2, PRPH2, SAG, SPTBN5	CC	3	9.09	6	<0.001	0.020
GO:0098813	nuclear chromosome segregation	DLGAP5, M1AP, MEIOC, NCAPD2, NCAPG, NUSAP1, SGO2, SLF1, SMC2, SMC5	BP	2	5.21	10	0.001	0.029
**List ii: Genes evolving under relaxed purifying or weak positive selection on the foreground (with ω0 < ω1 < 1), N* = 287 genes**								
GO:0007600	sensory perception	ABCA4, ABLIM1, ACPP, ARR3, ATP8B1, BEST1, CACNA2D4, CHRNA4, CNGB1, GABRR2, GRK7, GUCA1B, GUCY2F, IMPG1, IMPG2, LOXHD1, MMP24, MYO3A, MYO6, OPN1MSW, OTOF, PCDH15, PGAP1, RD3, RGS9, ROR1, RP1, RP1L1, RPE65, SCNN1B, TAS1R3, TBL1X, TMC2	BP	7	9.32	33	<0.001	<0.001
GO:0050877	nervous system process	ABCA4, ABLIM1, ACPP, ADCY5, ARR3, ATP8B1, BEST1, CACNA2D4, CHRNA4, CNGB1, DDHD2, DOP1B, GABRA6, GABRR2, GRIN2C, GRK7, GUCA1B, GUCY2F, IMPG1, IMPG2, KCNK2, LOXHD1, MMP24, MYO3A, MYO6, OPN1MSW, OTOF, PCDH15, PGAP1, PLA2G6, RD3, RGS9, RIMS2, ROR1, RP1, RP1L1, RPE65, SCNN1B, SHISA9, SLC6A4, TAS1R3, TBL1X, TMC2, UNC13B, ZMPSTE24	BP	7	6.61	45	<0.001	<0.001
GO:0007601	visual perception	ABCA4, ABLIM1, ARR3, BEST1, CACNA2D4, CNGB1, GABRR2, GRK7, GUCA1B, GUCY2F, IMPG1, IMPG2, MYO3A, OPN1MSW, PCDH15, RD3, RGS9, RP1, RP1L1, RPE65	BP	7	12.99	20	<0.001	<0.001
GO:0009584	detection of visible light	ABCA4, BEST1, CACNA2D4, CNGB1, GRK7, GUCA1B, GUCY2F, OPN1MSW, RP1, RPE65	BP	7	19.61	10	<0.001	<0.001
GO:0009581	detection of external stimulus	ABCA4, BEST1, CACNA2D4, CNGB1, GRK7, GUCA1B, GUCY2F, MMP24, OPN1MSW, PCDH15, RP1, RPE65, TMC2	BP	7	13.00	13	<0.001	<0.001

GO:0097733	photoreceptor cell cilium	ABCA4, ARR3, CEP290, CNGB1, CRY1, GRK7, GUCA1B, GUCY2F, OPN1MSW, PCDH15, RP1, RP1L1	CC	7	14.12	12	<0.001	<0.001
GO:0009582	detection of abiotic stimulus	ABCA4, BEST1, CACNA2D4, CNGB1, GRK7, GUCA1B, GUCY2F, MMP24, OPN1MSW, PCDH15, RP1, RPE65, TMC2	BP	7	12.62	13	<0.001	<0.001
GO:0097730	non-motile cilium	ABCA4, ANO2, ARR3, CEP290, CNGB1, CRY1, GRK7, GUCA1B, GUCY2F, OPN1MSW, PCDH15, RP1, RP1L1	CC	7	12.38	13	<0.001	<0.001
GO:0120025	plasma membrane bounded cell projection	ABCA4, ABLIM1, ACPP, ADCY5, ADCY9, ANO2, APPL2, ARHGEF4, ARR3, ATP6V1A, ATP8B1, CDK6, CEP170, CEP290, CHRNA4, CLIP1, CNGB1, CRY1, CTSZ, DAGLA, DNAH3, DNAH8, DNAH9, DNM1, DTNA, DYSF, GABRA6, GRK7, GUCA1B, GUCY2F, KCNK2, KIF4A, KLHL24, LAMA2, LAMP5, LOXHD1, LRP4, LRP8, MARK1, MYO3A, MYO6, NEK8, OPN1MSW, PCDH15, PDLIM5, PTPRS, ROR1, RP1, RP1L1, RPH3A, RTTN, SBF2, SHISA9, STAR, SYNE2, SYT11, TMC2, UNC13B, UNC13C, UNC5A, WRN	CC	6	4.16	61	<0.001	<0.001
GO:0097458	neuron part	ABCA4, ADCY9, ARR3, ATP8B1, CEL, CEP290, CHRNA4, CNGB1, CRY1, CTSZ, DAGLA, DNM1, DTNA, ENO2, GABRA6, GRK7, GUCA1B, GUCY2F, HSPA2, ITPR1, KCNK2, KIF4A, KLHL24, LAMA2, LAMP5, LOXHD1, LRP4, LRP8, MARK1, MYO3A, OPN1MSW, OTOF, PCDH15, PDLIM5, PTPRS, RGS9, RIMS2, ROR1, RP1, RP1L1, RPH3A, SBF2, SHISA9, SLC16A3, SLC40A1, SLC6A4, STAR, SYT11, TMC2, UNC13B, UNC13C, UNC5A, WRN	CC	6	4.38	53	<0.001	<0.001
GO:0043005	neuron projection	ABCA4, ADCY9, ARR3, ATP8B1, CEP290, CHRNA4, CNGB1, CRY1, CTSZ, DAGLA, DNM1, DTNA, GABRA6, GRK7, GUCA1B, GUCY2F, KCNK2, KIF4A, KLHL24, LAMA2, LAMP5, LOXHD1, LRP4, LRP8, MARK1, MYO3A, OPN1MSW, PCDH15, PDLIM5, PTPRS, ROR1, RP1, RP1L1, RPH3A, SBF2, SHISA9, STAR, SYT11, TMC2, UNC13B, UNC13C, UNC5A, WRN	CC	6	4.83	43	<0.001	<0.001
GO:0050906	detection of stimulus involved in sensory perception	BEST1, CACNA2D4, CNGB1, GUCY2F, MMP24, PCDH15, RPE65, TAS1R3, TMC2	BP	7	16.67	9	<0.001	0.001
KEGG:04744	Phototransduction	CNGB1, GRK7, GUCA1B, GUCY2F, OPN1MSW, RGS9	KEGG	7	26.09	6	<0.001	0.002
GO:0050908	detection of light stimulus involved in visual perception	BEST1, CACNA2D4, CNGB1, GUCY2F, RPE65	BP	7	35.71	5	<0.001	0.002
GO:0120038	plasma membrane bounded cell projection part	ABCA4, ADCY9, APPL2, ARHGEF4, ARR3, ATP8B1, CEP170, CEP290, CHRNA4, CNGB1, CRY1, CTSZ, DAGLA, DNAH3, DNAH8, DNAH9, DNM1, GABRA6, GRK7, GUCA1B, GUCY2F, KCNK2, KIF4A, LAMA2, LAMP5, LRP4, LRP8, MARK1, MYO3A, MYO6, NEK8, OPN1MSW, PCDH15, ROR1, RP1, RP1L1, RTTN, SHISA9, SYNE2, SYT11, TMC2, UNC13B, UNC13C, UNC5A	CC	6	4.32	44	<0.001	0.004
GO:0097381	photoreceptor disc membrane	ABCA4, CRY1, GRK7, GUCA1B, GUCY2F	CC	7	31.25	5	<0.001	0.005
GO:0007602	phototransduction	ABCA4, CNGB1, GRK7, GUCA1B, GUCY2F, OPN1MSW, RP1	BP	7	16.28	7	<0.001	0.010
GO:0016459	myosin complex	CGNL1, MYBPC1, MYH11, MYH15, MYL1, MYO3A, MYO6	CC	5	14.89	7	<0.001	0.018

GO:0001895	retina homeostasis	ABCA4, CNGB1, PCDH15, PRDX1, RP1, RP1L1, RPE65	BP	7	14.58	7	<0.001	0.021
GO:0007605	sensory perception of sound	ATP8B1, LOXHD1, MYO3A, MYO6, OTOF, PCDH15, PGAP1, ROR1, TBL1X, TMC2	BP	4	9.80	10	<0.001	0.022
GO:0042461	photoreceptor cell development	CEP290, CNGB1, PRDM1, RP1, RP1L1, RPE65	BP	7	17.65	6	<0.001	0.023
GO:0003774	motor activity	CGNL1, DNAH3, DNAH8, KIF15, KIF4A, MYH11, MYH15, MYO3A, MYO6	MF	5	10.84	9	<0.001	0.023
GO:0071103	DNA conformation change	ASH1L, ATRX, CHAF1A, FANCM, HP1BP3, MIS18BP1, MNAT1, NCAPH, RAD54B, SRPK1, TOP2A, TPR, WRN	BP	1	7.65	13	<0.001	0.027
GO:0005875	microtubule associated complex	ACTR10, DNAH3, DNAH8, KIF15, KIF4A, LRP8, RP1, TPR	CC	5	11.76	8	<0.001	0.031
GO:0099512	supramolecular fiber	AKAP13, ASPM, CCT6A, CDC27, CEP170, CKAP5, CLIP1, COL3A1, COL5A1, DNAH3, DNAH8, DNAH9, DNM1, DYSF, KIF4A, KRT5, MYBPC1, MYH11, MYH15, MYL1, MYO3A, MYO6, NEB, OBSL1, RP1, RP1L1, SYNE2	CC	2	4.73	27	<0.001	0.045
GO:1902495	transmembrane transporter complex	ANO2, ATP12A, BEST1, CACNA2D4, CHRNA4, CNGB1, GABRA6, GABRR2, GRIN2C, HSPA2, KCNG4, KCNK2, SCNN1B, SHISA9	CC	3	6.86	14	<0.001	0.047
**List iii: Genes evolving under positive selection on specific sites of the foreground branch, N* = 123 genes**								
GO:0015631	tubulin binding	CLIP1, GAS8, INO80, KIF20B, KIF4A, MAP7D3, NUSAP1, RP1, SAXO1	MF	2	4.25	9	<0.001	0.002
GO:0140014	mitotic nuclear division	INO80, KIF20B, KIF4A, MTBP, NUSAP1, SLF1, SMC2, SMC5	BP	4	4.00	8	0.001	0.006
GO:0000070	mitotic sister chromatid segregation	INO80, KIF4A, NUSAP1, SLF1, SMC2, SMC5	BP	4	5.22	6	0.001	0.007
GO:0036126	sperm flagellum	DNAH1, GAS8, SAXO1, TEKT4	CC	3	7.27	4	0.002	0.017
GO:0001578	microtubule bundle formation	CLIP1, DNAH1, GAS8, RP1	BP	1	6.45	4	0.003	0.026
GO:0045931	positive regulation of mitotic cell cycle	KIF20B, MTBP, NUSAP1, SLF1, SMC5	BP	4	4.46	5	0.004	0.039

* Number of genes after all filtering steps

Table S9. Overrepresented functional GO-terms of gene list i, ii, iii with family-wise error rate (FWER) < 0.05 identified by GOfunR based on 10000 permutations.

GO ID	GO Term	Ontology category	Raw p-Value	FWER corrected p-Value
**List i: Genes evolving under strong positive selection on the foreground (with ω0 ≤ 1 < ω1). N* = 199 genes**				
GO:0097733	photoreceptor cell cilium	CC	<0.001	0.002
GO:0097731	9+0 non-motile cilium	CC	<0.001	0.002
GO:0097730	non-motile cilium	CC	<0.001	0.009
GO:0030261	chromosome condensation	BP	<0.001	0.029
GO:0000796	condensin complex	CC	<0.001	0.038
GO:0007076	mitotic chromosome condensation	BP	<0.001	0.039
**List ii: Genes evolving under relaxed purifying or weak positive selection on the foreground (with ω0 < ω1 < 1), N* = 287 genes**				
GO:0007600	sensory perception	BP	<0.001	<0.001
GO:0050877	nervous system process	BP	<0.001	<0.001
GO:0003008	system process	BP	<0.001	<0.001
GO:0007601	visual perception	BP	<0.001	<0.001
GO:0050953	sensory perception of light stimulus	BP	<0.001	<0.001
GO:0009584	detection of visible light	BP	<0.001	<0.001
GO:0001750	photoreceptor outer segment	CC	<0.001	<0.001
GO:0097733	photoreceptor cell cilium	CC	<0.001	<0.001
GO:0097731	9+0 non-motile cilium	CC	<0.001	<0.001
GO:0097730	non-motile cilium	CC	<0.001	<0.001
GO:0120025	plasma membrane bounded cell projection	CC	<0.001	0.001
GO:0009581	detection of external stimulus	BP	<0.001	0.001
GO:0043005	neuron projection	CC	<0.001	0.001
GO:0097458	neuron part	CC	<0.001	0.001
GO:0009582	detection of abiotic stimulus	BP	<0.001	0.001
GO:0042995	cell projection	CC	<0.001	0.002
GO:0009583	detection of light stimulus	BP	<0.001	0.002
GO:0097381	photoreceptor disc membrane	CC	<0.001	0.007
GO:0050906	detection of stimulus involved in sensory perception	BP	<0.001	0.009
GO:0044463	cell projection part	CC	<0.001	0.009
GO:0120038	plasma membrane bounded cell projection part	CC	<0.001	0.009
GO:0098590	plasma membrane region	CC	<0.001	0.011
GO:0042623	ATPase activity, coupled	MF	<0.001	0.021
GO:0016887	ATPase activity	MF	<0.001	0.022
GO:0016459	myosin complex	CC	<0.001	0.026
GO:0050908	detection of light stimulus involved in visual perception	BP	<0.001	0.028
GO:0050962	detection of light stimulus involved in sensory perception	BP	<0.001	0.028
GO:0003774	motor activity	MF	<0.001	0.037
GO:0005875	microtubule associated complex	CC	<0.001	0.049
**List iii: Genes evolving under positive selection on specific sites of the foreground branch, N* = 123 genes**				
GO:0008017	microtubule binding	MF	<0.001	0.016
GO:0070701	mucus layer	CC	<0.001	0.035

CC=cellular_component, BP=biological_process, MF=molecular_function

* Number of genes after all filtering steps
